# Comparison of the dynamics of neural interactions between current-based and conductance-based integrate-and-fire recurrent networks

**DOI:** 10.3389/fncir.2014.00012

**Published:** 2014-03-05

**Authors:** Stefano Cavallari, Stefano Panzeri, Alberto Mazzoni

**Affiliations:** ^1^Center for Neuroscience and Cognitive Systems@UniTn, Istituto Italiano di TecnologiaRovereto, Italy; ^2^Max Planck Institute for Biological CyberneticsTübingen, Germany; ^3^The BioRobotics Institute, Scuola Superiore Sant'AnnaPisa, Italy

**Keywords:** recurrent neural network, integrate-and-fire neurons, current based neuron models, conductance based neuron models, spike correlation, local field potentials, correlation analysis, information encoding

## Abstract

Models of networks of Leaky Integrate-and-Fire (LIF) neurons are a widely used tool for theoretical investigations of brain function. These models have been used both with current- and conductance-based synapses. However, the differences in the dynamics expressed by these two approaches have been so far mainly studied at the single neuron level. To investigate how these synaptic models affect network activity, we compared the single neuron and neural population dynamics of conductance-based networks (COBNs) and current-based networks (CUBNs) of LIF neurons. These networks were endowed with sparse excitatory and inhibitory recurrent connections, and were tested in conditions including both low- and high-conductance states. We developed a novel procedure to obtain comparable networks by properly tuning the synaptic parameters not shared by the models. The so defined comparable networks displayed an excellent and robust match of first order statistics (average single neuron firing rates and average frequency spectrum of network activity). However, these comparable networks showed profound differences in the second order statistics of neural population interactions and in the modulation of these properties by external inputs. The correlation between inhibitory and excitatory synaptic currents and the cross-neuron correlation between synaptic inputs, membrane potentials and spike trains were stronger and more stimulus-modulated in the COBN. Because of these properties, the spike train correlation carried more information about the strength of the input in the COBN, although the firing rates were equally informative in both network models. Moreover, the network activity of COBN showed stronger synchronization in the gamma band, and spectral information about the input higher and spread over a broader range of frequencies. These results suggest that the second order statistics of network dynamics depend strongly on the choice of synaptic model.

## Introduction

Networks of Leaky Integrate-and-Fire (LIF) neurons are a key tool for the theoretical investigation of the dynamics of neural circuits. Models of LIF networks express a wide range of dynamical behaviors that resemble several of the dynamical states observed in cortical recordings (see Brunel, 2013 for a recent review). An advantage of LIF networks over network models that summarize neural population dynamics with only the density of population activity, such as neural mass models (Deco et al., 2008), is that LIF networks include the dynamics of individual neurons. Therefore LIF networks can be used to investigate phenomena, such as the relationships among spikes of different neurons, that are not directly accessible to simplified mass models of network dynamics.

A basic choice when designing a LIF network is whether the synaptic model is voltage-dependent (conductance-based model) or voltage-independent (current-based model). In the former case the synaptic current depends on the driving force, while this does not happen in the current-based model. Current-based LIF models are popular because of their relative simplicity (see e.g., Brunel, 2013) and they have the key advantage of facilitating the derivation of analytical closed-form solutions. Thus current-based synapses are convenient for developing mean field models (Grabska-Barwinska and Latham, 2013), event-based models (Touboul and Faugeras, 2011), or firing rate models (Helias et al., 2010; Ostojic and Brunel, 2011; Schaffer et al., 2013), as well as in studies examining the stability of neural states (Babadi and Abbott, 2010; Mongillo et al., 2012). Moreover, current-based models are often adopted, because of their simplicity, to investigate numerically network-scale phenomena (Memmesheimer, 2010; Renart and Van Rossum, 2012; Gutig et al., 2013; Lim and Goldman, 2013; Zhang et al., 2013). On the other hand, conductance-based models are also widely used because they are more biophysically grounded (Kuhn et al., 2004; Meffin et al., 2004). In particular, only conductance-based neurons can reproduce the fact that when the synaptic input is intense, cortical neurons display a three- to fivefold decrease in membrane input resistance (thus they enter a high-conductance state), as observed in intracellular recordings *in vivo* (Destexhe et al., 2003). However, an added complication of conductance-based models is that their differential equations can only be evaluated numerically or approximated analytically (Rudolph-Lilith et al., 2012) rather than being fully analytically treatable.

Despite the widespread use of both types of models, the differences in the network dynamics that they generate has not been yet fully understood. Previous studies comparing conductance- and current-based LIF models focused mostly on the individual neuron dynamics (Kuhn et al., 2004; Meffin et al., 2004; Richardson, 2004). Here we extended these previous works by investigating the network level consequences of the synaptic model choice. In particular, we investigated which aspects of network dynamics are independent of the choice of the specific synaptic model, and which are not. Understanding this point is crucial for fully evaluating the costs and implications of adopting a specific synaptic model.

We compared the dynamics of two sparse recurrent excitatory-inhibitory LIF networks, a conductance-based network (COBN) with conductance-based synapses, and a current-based network (CUBN) with current-based synapses. To properly compare the two networks, we set to equal values all the common parameters (including the connectivity matrix). Building on previous works (La Camera et al., 2004; Meffin et al., 2004), we devised a novel algorithm to obtain two comparable networks by properly tuning the synaptic conductance values of the COBN given the set of values of synaptic efficacies of the CUBN. Since the differences between the dynamics of the two synaptic models depend on the fluctuations of the driving force (i.e., of the membrane potential), they should be close to zero when the synaptic activity is low. Thus, when decreasing the background synaptic activity, the Post-Synaptic Currents (PSCs) of the two models should become more and more similar. Consequently, our procedure calibrated the conductances so that PSCs became exactly equal in the limit of zero synaptic input (see Methods). Then we investigated whether this procedure could generate COBNs and CUBNs with matching average single neuron stationary firing rates under a reasonably wide range of parameters and network stimulation conditions. We then studied how comparable conductance- and current-based networks differed in more complex characterizations of population dynamics, such as the cross-neuron correlations of membrane potential (MP), input current and spike train, as well as the spectrum of network fluctuations. The latter was investigated not only for total average firing rates, but also for the simulated Local Field Potential (LFP) computed from the massed synaptic activity of the networks (Mazzoni et al., 2008). To study the spectrum of network fluctuations it is useful to use a LFP model (rather than a massed spike rate) mainly because cortical rhythms are more easily measured in experiments by recording LFPs rather than the spike rate (Buzsaki et al., 2012; Einevoll et al., 2013); therefore this quantification makes the models more directly comparable to experimental observations. We then quantified how the external inputs modulate the firing rate, the LFP spectrum and the spike train correlation by using information theory (Quian Quiroga and Panzeri, 2009; Crumiller et al., 2011). Finally, we discuss the similarities and differences of COBN and CUBN against recent experimental observations of dynamics of cortical network correlations (Lampl et al., 1999; Kohn and Smith, 2005; De La Rocha et al., 2007; Okun and Lampl, 2008; Ecker et al., 2010; Renart et al., 2010).

## Methods

### Network structure and external inputs

We considered two networks of LIF neurons with identical architecture and injected with identical external inputs. The only difference between the two networks was in the synaptic model: one was composed by neurons with conductance-based synapses and the other by neurons with current-based synapses (see subsection “Single neuron models” in Methods). The network structure was the same one used in a previous work (Mazzoni et al., 2008), to which we refer for a full description. Briefly, each network was composed of 5000 neurons. Eighty percent of the neurons were excitatory, that is their projections onto other neurons formed AMPA-like excitatory synapses, while the remaining 20% were inhibitory, that is their projections formed (A-type) GABA-like inhibitory synapses. The 4:1 ratio is compatible with anatomical observations (Braitenberg and SchüZ, 1991). The network had random connectivity with a probability of directed connection between each pair of neurons of 0.2 (Sjostrom et al., 2001; Holmgren et al., 2003), thus any neuron in the network received on average 200 synaptic contacts from inhibitory neurons and 800 from excitatory neurons (see Supplementary Figure 1). Both populations received a noisy excitatory external input taken to represent the activity from thalamocortical afferents, with inhibitory neurons receiving stronger inputs than excitatory neurons. This simulated external input was implemented as a series of spike times that activated excitatory synapses with the same kinetics as recurrent AMPA synapses, but different strengths (see Tables 1, 2).

**Table 1:** **Synaptic efficacies of the current-based network**.

**Current-based network**	
**SYNAPTIC EFFICACIES, *J*_syn_ (pA)**	
GABA on inhibitory	54
GABA on excitatory	42.5
AMPA_recurrent_ on inhibitory	−14
AMPA_recurrent_ on excitatory	−10.5
AMPA_external_ on inhibitory	−19
AMPA_external_ on excitatory	−13.75

**Table 2:** **Reference values of the synaptic parameters of the conductance-based model**.

**Conductance-based network**	
**SYNAPTIC CONDUCTANCES, *g*_syn_ (nS)**	
GABA on inhibitory	2.70
GABA on excitatory	2.01
AMPA_recurrent_ on inhibitory	0.233
AMPA_recurrent_ on excitatory	0.178
AMPA_external_ on inhibitory	0.317
AMPA_external_ on excitatory	0.234
**SYNAPTIC REVERSAL POTENTIAL, *V*_syn_ (mV)**	
V_GABA_	−80
V_AMPA_	0

The input spike trains activating the model thalamocortical synapses were generated by a Poisson process, with a time-varying rate, ν_ext_(*t*), identical for all neurons. Note that this implied that the variance of the inputs across neurons increased with the input rate. ν_ext_(*t*) was given by the positive part of the superposition of a “signal,” ν_signal_(*t*), and a “noise” component, *n*(*t*):

(1) $$\nu_{\text{ext}}(t)={\left[\nu_{\text{signal}}(t)+n(t)\right]}_{+}$$

The separation of signal and noise in the input spike rate was to reproduce the classical experimental design in which a given sensory stimulus is presented many times, with each presentation (or “trial”) eliciting different responses due to variations in intrinsic network dynamics from presentation to presentation. We achieved this by identifying the external stimulus with the signal term, ν_signal_(*t*), (which was thus exactly the same across all trials of the same stimulus) and by using a noise term, *n*(*t*), generated (as explained below) independently in each trial.

In this study we used three kinds of external signals. For the majority of the simulations we used constant stimuli, ν_signal_(*t*) = ν_0_, (with ν_0_ ranging from 1.5 to 6 spikes/ms). In a second set of simulations we used periodic stimuli made by superimposing a constant baseline term to a sinusoid: ν_signal_(*t*) = *A* sin(2π *ft*) + ν_0_, where *A* = 0.6 spikes/ms; *f* ranged from 2 to 16 Hz in Figure 12 and from 2 to 150 Hz in Figure 13 and ν_0_ was set to 1.5 (respectively 5) spikes/ms when studying the low- (respectively high-) conductance state. We also used a time-varying signal that reproduced the time course of Multi Unit Activity recorded from the LGN of an anaesthetized macaque during binocular presentation of commercially available color movies (Belitski et al., 2008). This latter dynamical stimulus, called “naturalistic”, is fully described and characterized in (Mazzoni et al., 2008) to which we refer for further details. For the purposes of the present work, it is useful to remind that this naturalistic signal was a slow signal dominated by frequencies below 4 Hz.

The noise component of the stimuli, *n*(*t*), was generated by an Ornstein-Uhlenbeck (OU) process with zero mean:

(2) $$\tau_{n}\frac{dn(t)}{dt}=-n(t)+\sigma_{n}(\sqrt{2\tau_{n}})\eta (t),$$

where σ^2^_*n*_ = 0.16 spikes/ms is the variance of the noise, and η(*t*) is a Gaussian white noise. The time constant τ_*n*_ was set to 16 ms to have a cut-off frequency of 10 Hz. Note that the trial-to-trial differences in the stochastic process generated by Equation 2 were the first and largest source of trial-to-trial variability in the model, the second and last being the fact that each neuron received an independent realization of the Poisson process with rate ν_ext_(*t*).

In a specific set of control stimulations (Supplementary Figure 4), instead of the OU process described above, we used a Gaussian white noise with the same variance. Note that, for low frequencies, the power spectrum of the OU process was higher than the one of the white noise.

### Single neuron models

Both inhibitory and excitatory neurons were modeled as LIF neurons (Tuckwell, 1988). The leak MP, *V*_leak_, was set to −70 mV, the spike threshold, *V*_threshold_, to −52 mV and the reset potential, *V*_reset_, to −59 mV. The absolute refractory period was set to 2 ms for excitatory neurons and to 1 ms for inhibitory neurons (Brunel and Wang, 2003). The equation for the sub-threshold dynamic of the MP of i-th neuron had the following form:

(3) $$\tau_{m}\frac{dV^{i}(t)}{dt}=-V^{i}(t)+V_{\text{leak}}-\frac{I_{\text{tot}}^{i}(t)}{g_{\text{leak}}},$$

where τ_*m*_ is the membrane time constant (20 and 10 ms for excitatory and inhibitory neurons respectively), *g*_leak_ is the leak membrane conductance (25 nS and 20 nS for excitatory and inhibitory neurons respectively) (Brunel and Wang, 2003) and *I*^*i*^_tot_ (*t*) is the total synaptic input current. The latter was given by the sum of all the synaptic inputs entering the i-th neuron:

(4) $$I_{\text{tot}}^{i}(t)=\sum_{N_{\left(\text{i},\text{ AMPArec}\right)}}I_{\text{AMPArec}}^{i}(t)+\sum_{N_{\left(\text{i},\text{ GABA}\right)}}I_{\text{GABA}}^{i}(t)+I_{\text{AMPAext}}^{i}(t),$$

the value of *N*_(i, AMPArec)_ (respectively *N*_(i, GABA)_) being the set of excitatory (respectively inhibitory) neurons projecting into the i-th neuron, and *I*^*i*^_AMPArec_(*t*), *I*^*i*^_GABA_(*t*), *I*^*i*^_AMPAext_(*t*) the different synaptic inputs entering the *i*-th neuron from: recurrent AMPA, GABA, and external AMPA synapses respectively.

The difference between current- and conductance-based synapses lied in the definition of these synaptic input currents *I*_syn_. For the current-based model:

(5) $$I_{\text{syn}}^{\text{CUBN}}(t)=J_{\text{syn}}s_{\text{syn}}(t),$$

where *J*_syn_ are the synaptic efficacies (see Table 1) and *s*_syn_(*t*) a function that models the synaptic kinetics (see below).

In the conductance-based model the synaptic input currents depended also on the MP, *V*(*t*):

(6) $$I_{\text{syn}}^{\text{COBN}}(t)=g_{\text{syn}}s_{\text{syn}}(t)(V(t)-V_{\text{syn}}),$$

where *g*_syn_ and *V*_syn_ are respectively the conductance and the reversal potential of the synapse; the term (*V*(*t*) − *V*_syn_) is the driving force of the synaptic current. The values of the parameters *g*_syn_ in Equation 6 were computed as described in the subsection “Procedure to determine comparable COBN and CUBN models.” The reference values of reversal potentials and synaptic conductances are displayed in Table 2. In Figures 6C,D and 7D these values were varied to test the robustness of our results.

The same function *s*_syn_(*t*) described the time course of the synaptic currents in both models; it depended both on the synapse type and on the kind of neuron receiving the input. Every time a pre-synaptic spike occurred at time t^*^, *s*_syn_(*t*) of the post-synaptic neuron was incremented by an amount described by a delayed difference of exponentials (Brunel and Wang, 2003):

(7) $$\Delta s_{\text{syn}}(t)=\frac{\tau_{m}}{\tau_{d}-\tau_{r}}\left[\exp\text{​}\left(-\frac{t-\tau_{l}-t^{*}}{\tau_{d}}\right)-\exp\left(-\frac{t-\tau_{l}-t^{*}}{\tau_{r}}\right)\right],$$

where the latency τ_*l*_, the rise time τ_*r*_ and the decay time τ_*d*_ are shown in Table 3.

**Table 3:** **Synaptic time constants of both models**.

**Synaptic time constants (ms)**	**τ_l_**	**τ_r_**	**τ_d_**
GABA	1	0.25	5
AMPA on inhibitory	1	0.2	1
AMPA on excitatory	1	0.4	2

A useful parameter for conductance-based neuron analysis is the effective membrane time constant τ_eff_. Following a standard procedure we computed the total effective membrane conductance for the *i*-th neuron as:

(8) $$\begin{array}{l} g_{\text{tot}}^{i}(t)=g_{\text{leak}}+\sum_{N(\text{i},\text{ AMPArec})}g_{\text{AMPArec}}s_{\text{AMPArec}}^{i}(t)\\ \;+\sum_{N(\text{i},\text{ GABA})}g_{\text{GABA}}s_{\text{GABA}}^{i}\text{​}(t)+g_{\text{AMPAext}}s_{\text{AMPAext}}^{i}(t), \end{array}$$

and we rewrote Equation 3 as follows:

(9) $$\tau_{\text{eff}}^{i}(t)\frac{dV^{i}(t)}{dt}=-V^{i}(t)+\frac{g_{\text{leak}}V_{\text{leak}}+\sum_{N(\text{i},\text{ syn})}g_{\text{syn}}s_{\text{syn}}^{i}(t)V_{\text{syn}}}{g_{\text{tot}}^{i}(t)}$$

(10) $$\text{where }\tau_{\text{eff}}^{i}(t)=\frac{\tau_{m}g_{\text{leak}}}{g_{\text{tot}}^{i}(t)}$$

is the effective membrane time constant and “syn” indicates: recurrent AMPA; GABA; external AMPA. In particular, for the *i*-th neuron, the effective AMPA conductance is defined as ∑_*N*(i, AMPArec)_*g*_AMPArec_ *s*^*i*^_AMPArec_(*t*) + *g*_AMPAext_ *s*^*i*^_AMPAext_(*t*) and the effective GABA conductance as ∑_*N*(i, GABA)_*g*_GABA_ *s*^*i*^_GABA_(*t*) (see Figure 3).

### Numerical methods

Network simulations were done using a finite difference integration scheme based on the second-order Runge Kutta algorithm (Press et al., 1992), also known as the midpoint method, with time step Δ*t* = 0.05 ms.

The noise, *n*(*t*), was obtained from Equation 2 by implementing an exact numerical simulation of the Ornstein-Uhlenbeck process (Gillespie, 1996). The temporal durations of the simulations varied from 4.5 s to 100.5 s, and they are specified in the figure captions. The regimes we investigated displayed average firing rates relatively low (0.4–13 Hz), thus, when computing the Inter-Spike Interval (ISI) and the pairwise spike train correlation, we used the longest simulation times (25.5 and 100.5 s) to obtain larger spike datasets. Since we studied stationary responses, the first 500 ms of the simulations were never included in any analysis. Analysis and simulations (the latter implemented using MEX file) were performed in Matlab. Both COBN and CUBN model source codes are available as Supplemental Material to this paper and on the ModelDB sharing repository (http://senselab.med.yale.edu/ModelDB/ShowModel.asp?model=152539) with accession number 152539.

### Spectral analysis

To compute the power spectrum we used the Fast Fourier Transform with the Welch method (pwelch function in Matlab), dividing the time window under investigation into eight subwindows with 50% overlap.

For the entrainment analysis showed in Figure 13 in case of periodic inputs with frequency *f*, we bandpassed the LFP at the correspondent frequency *f* with a Kaiser filter with zero phase lag and 2 Hz bandwidth, very small passband ripple (0.05 dB) and high stopband attenuation (60 dB). We extracted then the instantaneous phase by means of the Hilbert transform of the signal. To quantify entrainment, we computed the phase coherence between the phase of the input signal and of the LFP at the corresponding frequency (Mormann et al., 2000). Phase coherence, which we computed using the CircStat toolbox (Berens, 2009), ranges from zero (no relationships between phases) to 1 (perfect phase locking between the two signals).

### Computation of simulated local field potential

We computed from network activity the LFP by using a procedure that has been proposed in previous works (Mazzoni et al., 2008, 2010, 2011), to which we refer for full details. The procedure is summarized and motivated in the following. LFPs are experimentally obtained by low-pass filtering the extracellularly recorded neural signal, and are thought to reflect to a first approximation the current flow due to synaptic activity around the tip of the recording electrode (Buzsaki et al., 2012). Thus, we computed the simulated LFP as the difference between the sum of the GABA currents and the sum of the AMPA currents (both external and recurrent) that enter all excitatory neurons. This quantity was then divided by the leak membrane conductance to obtain units of mV.

This simple recipe was motivated by two well-known geometrical properties of cortical circuits. First, AMPA synapses tend to be apical, i.e., they contact the dendrites away from the soma, while GABA synapses tend to be peri-somatic, i.e., they contact the soma or the dendrites close to the soma. Because of this spatial arrangement, the sink and sources resulting from the activation of both AMPA and GABA synapses will tend to produce in the extracellular field a dipole oriented from apical dendrites toward soma; hence we computed the LFP by subtracting the AMPA currents from the GABA currents (divided by the leak membrane conductance). Second, pyramidal neurons contribute more than interneurons to generation of LFPs in cortex because their apical dendrites are organized in an approximate open field configuration, whereas the organization of dendrites of interneurons is arranged to a first approximation in a close field configuration (Lorente De No, 1947; Murakami and Okada, 2006; Linden et al., 2011). Therefore we computed LFPs by considering only input currents to excitatory neurons (taken here to correspond to cortical pyramidal neurons). This model, though simple, proved to be an effective way to generate a realistic LFP signal that match many characteristics of LFPs in sensory cortex (Mazzoni et al., 2008, 2010, 2011).

### Procedure to determine comparable current- and conductance-based networks

As mentioned above all the parameters that were directly shared between the two models were set equal; also the connectivity matrix was the same in the CUBN and in the COBN. The starting point of our comparison was to completely define the CUBN, by specifying the synaptic efficacies, *J*_syn_ (reported in Table 1), as well as the values of the common set of parameters. Then, we computed the synaptic parameters of the COBN that made it comparable to the given CUBN. To simplify the problem, we first set the reversal potentials of the COBN to biophysically plausible values: *V*_AMPA_ = 0 mV and *V*_GABA_ = −80 mV (as reference values, but we also tested other values, see Figures 6C,D, 7D). The “free” parameters left to set were now only the COBN conductances (*g*_syn_ in Equation 6).

**Figure 1:**
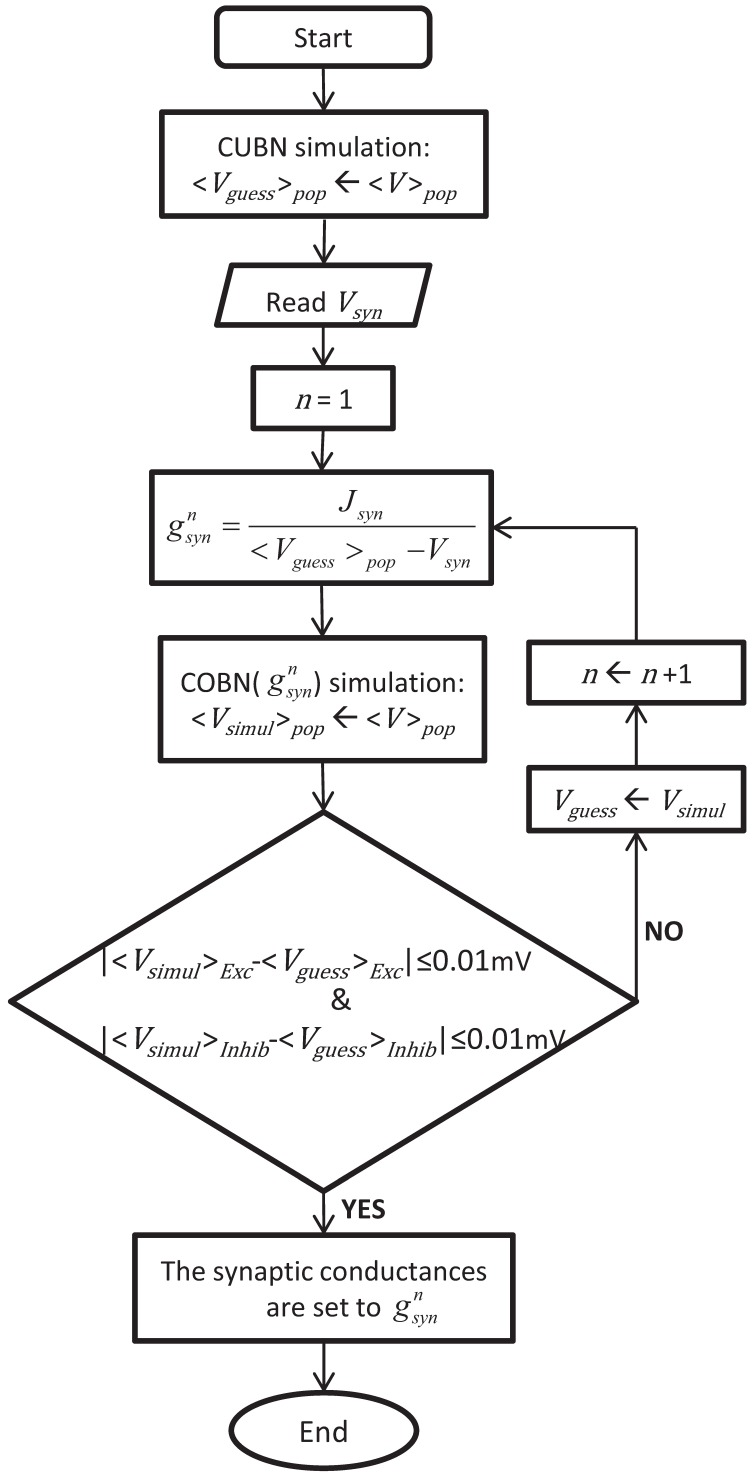
**Procedure to set the synaptic conductances of the COBN**. The flowchart illustrates the iterative algorithm we used to set the synaptic conductances, *g*_syn_, such in a way to obtain a COBN comparable with the given CUBN. The two networks shared all the common parameters, so, once the CUBN was given, the synaptic conductances depended only on the synaptic reversal potentials of the COBN, *V*_syn_.

The procedure used to obtain the conductance values leading to comparable COBN and CUBN is illustrated in Figure 1 and described in the following. Consistent with the fact that the effective membrane time constant of the COBN is equal to the membrane time constant of the CUBN only in absence of synaptic input (see Equation 10), we set the conductances of each synapse type to obtain the same PSCs as in the corresponding current-based synapse in the limit of no synaptic activity. Explicitly, for each synapse type:

(11) $$g_{\text{syn}}=\frac{J_{\text{syn}}}{\left({\langle V\rangle }_{\text{pop}}-V_{\text{syn}}\right)},$$

where 〈*V*〉_pop_ was the average (over time and neurons) MP of excitatory and inhibitory populations obtained from network simulation of 4.5 s with a constant external input of 1.5 (spikes/ms)/cell. This last value was chosen because it was the lowest stimulus used throughout the paper, i.e., the one that induced the lowest synaptic activity. Since 〈*V*〉_pop_ depended on *g*_syn_, we determined both values numerically and recursively. We used as first guess the average MP obtained with the CUBN, we computed the associated conductances with Equation 11, we ran a COBN simulation with those conductances and then we used the resulting 〈*V*〉_pop_ to compute the updated conductances, until 〈*V*〉_pop_ (and consequently the conductances) reached a stable value (see Figure 1). Note that convergence was very fast: stability within a tolerance on average MPs of 0.01 mV was achieved usually in less than 10 steps. By using Equation 11, we rewrote the Equation 6 as follows:

(12) $$I_{\text{syn}}^{\text{COBN}}(t)=J_{\text{syn}}s_{\text{syn}}(t)\text{ ​}\left[1+\frac{V(t)-{\langle V\rangle }_{\text{pop}}}{{\langle V\rangle }_{\text{pop}}-V_{\text{syn}}}\right].$$

Comparing Equation 12 with Equation 5 it is clear that the synaptic currents of the two networks are the same only when *V*(*t*) = 〈*V*〉_pop_, that is in the limit of no synaptic input.

Conductance-based neurons can undergo transitions from low- to high-conductance states (Destexhe et al., 2001) and the simulations performed in this work included both states. However, current-based neurons cannot undergo such transitions and their membrane time constant is close to the effective membrane time constant of conductance-based neurons in a low-conductance state (see Figure 3A). Therefore, the correspondence between the two models that we defined is consistent with the physiologically-meaningful requirement that the differences between the two synaptic models decrease with synaptic activity (Destexhe et al., 2003).

### Computation of the average post-synaptic potentials in the conductance-based network

Modeling the synaptic input as conductance transients produces an activity-dependent increase of membrane conductance (that is a reduction of effective membrane time constant, see Equation 10) which attenuates and shortens the Post-Synaptic Potentials (PSPs) (Destexhe and Pare, 1999). In order to extract the average (activity-dependent) PSPs of the COBN we used a procedure similar to the one used in (Kumar et al., 2008): for each synapse type (see Table 2) we randomly selected 300 neurons from the network and we made a copy of them. These “cloned” neurons received the synaptic input of the original ones and had exactly the same spiking activity. The only difference with respect to the original is that the cloned neurons received an extra spike, from the synapse under investigation, each 100 ms (except for the first 500 ms), for a total of 100 PSPs for each cloned neuron (i.e., simulations lasted 10.5 s). We subtracted then the MP of the original neurons from the one of the cloned neurons and, by doing a spike triggered average over time and selected neurons, we obtained the average effective PSP.

### Computation of correlations among signals in the networks

We quantified the effects of the choice of the synaptic model on the cross-neuron correlation in time. We computed the cross-neuron pairwise Pearson's correlation coefficient of the time course of AMPA currents and of GABA currents entering the neurons, MPs and spike trains. The spike trains were binned in non-overlapping time windows of 5 ms and their correlation coefficients were averaged over all neuron pairs of the network (Figures 10A–C). Time courses of the other variables were expressed with the original time steps of 0.05 ms and the correlation was estimated averaging the correlation coefficients over all neurons' pairs obtained from two randomly selected subpopulations of 200 excitatory and 200 inhibitory neurons (Figure 9).

We measured also the average correlation between the time course of AMPA and GABA currents entering each single neuron. In particular, we computed the normalized cross-correlation between AMPA and GABA currents entering each neuron belonging to the two subpopulations of 200 neurons above mentioned. Then we averaged (over the neurons) the peak value and the peak position, i.e., the time lag for which the correlation was strongest (Figure 8).

### Computation of information about the external inputs

We studied how networks encoded external stimuli by means of mutual information between stimulus and response (that we will simply call information in the manuscript) (Shannon, 1948). The information that a set of responses, *R*, carries about a set of stimuli, *S*, is given by:

(13) $$I\text{​}\left(S;R\right)=\sum_{s\in S}P\text{​}(s)\sum_{r\in R}P\text{​}\left(r\text{|}s\right){\text{log}}_{2}\frac{P(r|s)}{P(r)},$$

where *P*(*s*) is the probability of presentation of the stimulus *s*, *P*(*r*) the probability of observing the response *r*, and *P*(*r|s*) the probability of observing *r* when *s* is presented.

**Figure 2:**
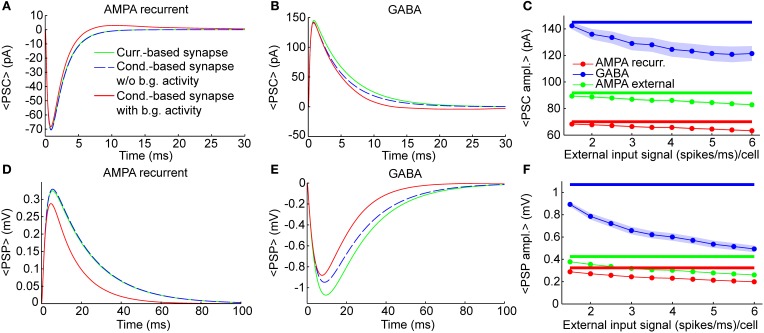
**Individual synaptic events**. Dynamics of single synaptic events on excitatory neurons (see Methods). Results were qualitatively very similar when considering synaptic inputs impinging on inhibitory neurons (see “PSP peak amplitude” in Supplementary Table 1). **(A,B)** Shape of Post-synaptic Currents (PSCs, top) for individual synaptic events in case of recurrent AMPA **(A)** and GABA **(B)** connection (thalamic AMPA case is not shown because it is qualitatively very similar to the recurrent AMPA case). The origin of the time axis corresponds to the arriving time of the spike. Green lines represent the kinetics in current-based neurons, which is independent from background synaptic activity. Dashed blue lines indicate the kinetics of an isolated conductance-based neuron (thus without background activity), having starting membrane potential equal to 〈*V*〉_exc_ = −58.8 mV, that is the average potential of the excitatory neurons of the network when the external input signal is 1.5 (spikes/ms)/cell. Red lines indicate the average PSCs in conductance-based neurons embedded in the network (thus with background activity) when the external input signal is 1.5 (spikes/ms)/cell (see Methods for details). Blue and green lines are superimposed in **(A)**. **(C)** Absolute average values of the PSC peaks as a function of the external input rate for neurons embedded in the network. Results are relative to recurrent AMPA (red) external AMPA (green), and GABA (blue) synapses for current- (thick lines) and conductance-based (thin lines with markers) neurons. Shaded areas for the conductance-based neurons correspond to the standard deviation across neurons (for AMPA connections the shaded areas are not visible because they are too small). **(D–F)** Same as **(A–C)** for Post-Synaptic Potentials (PSPs). PSPs are more relatively affected by the choice of the synaptic model with respect to the PSCs, because, in the COBN, the PSCs depend on the driving force, while the PSPs both on the driving force and on the effective membrane time constant.

**Figure 3:**
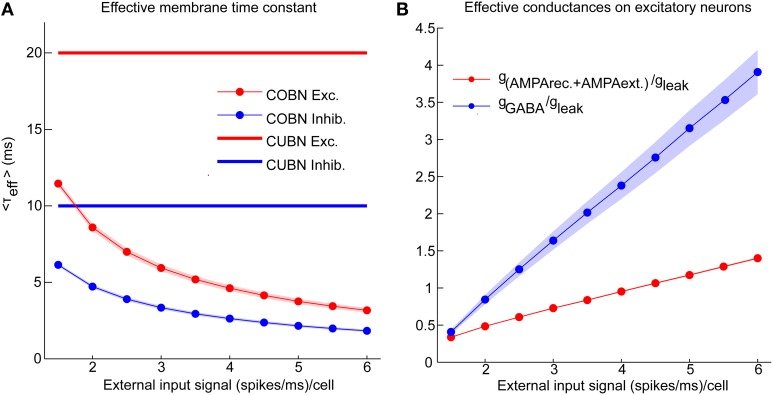
**Effective parameters in conductance-based networks**. Input rate modulations of COBN-specific parameters. **(A)** Average effective membrane time constant for conductance-based excitatory neurons (red markers) and inhibitory neurons (blue markers) as a function of the external input rate. Membrane time constants of the current-based neurons are shown for reference as thick lines. Results show that conductance-based membrane timescale is much faster than current-based one and that it decreases with input strength. **(B)** Average effective AMPA (red) and GABA (blue) conductances on excitatory neurons as a function of the external input rate. Results show that the COBN goes from low- to high-conductance states in the range of external stimuli considered. Same color code as **(A)**. Shaded areas represent standard deviation across neurons [in **(A)** for inhibitory time constant and in **(B)** for AMPA conductances they are not visible because too small]. Values are computed from a simulation of 10.5 s per stimulus and are averaged over time and neurons.

**Figure 4:**
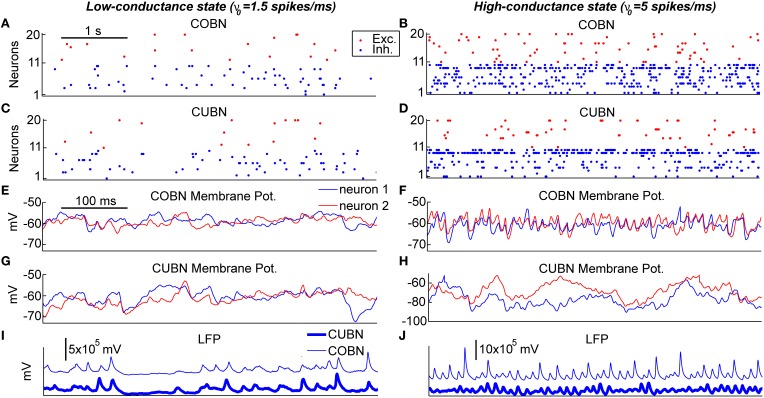
**Example traces**. Examples of 5 s **(A–D)** and 500 ms **(E–J)** of data traces generated by the two networks when using constant stimuli. The left column shows the activity in response to an input rate ν_0_ set to 1.5 spikes/ms generating a low-conductance state. The right column shows the activity in response to an input rate ν_0_ set to 5 spikes/ms generating a high-conductance state. **(A–D)** Raster plot of 10 excitatory and 10 inhibitory neurons taken from the COBN **(A,B)** and from the CUBN **(C,D)**. The selected neurons and the color code are the same across panels **(A–D)**. **(E–H)** Membrane potential of two neurons taken from the COBN **(C,D)** and from the CUBN **(G,H)**. The neurons displayed and the color code are the same across the panels **(E–H)**. **(I,J)** Simulated LFP obtained from the COBN (thin line) and from the CUBN (thick line).

**Figure 5:**
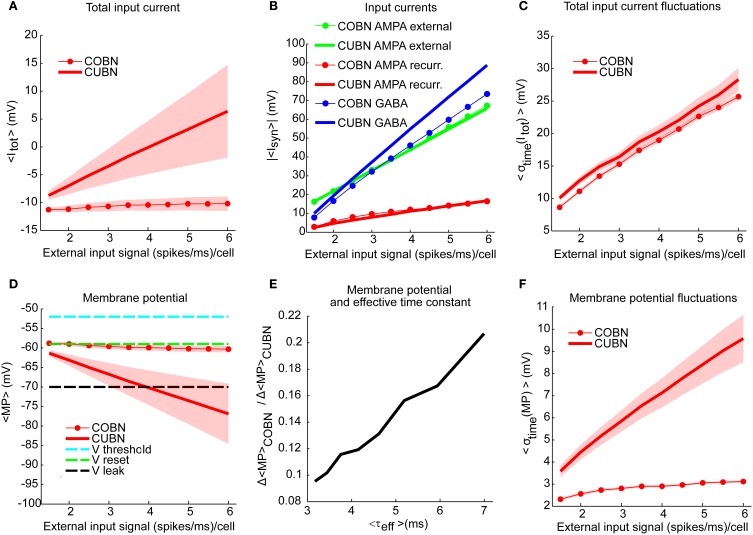
**Membrane potential and synaptic input currents as a function of the external input rate**. Effects of external input rate modulation on the net synaptic input currents and the membrane potential of excitatory neurons. The synaptic currents in panels **(A–C)** are divided by the leak membrane conductance to obtain units of mV. Results are qualitatively very similar when considering inhibitory neurons [see “MP” and “σ_time_ (MP)” in Supplementary Table 1]. We studied separately the average over time and the standard deviation over time of the variables by using a simulation of 10.5 s per stimulus. Shaded areas correspond to standard deviation across neurons. **(A)** Average total synaptic input current in CUBN (thick line) and COBN (thin line with markers) as a function of the external input rate. **(B)** Different input currents in the two networks. Blue/red/green lines represent respectively the average GABA/recurrent AMPA/external AMPA currents in CUBN (thick lines) and in COBN (thin lines with markers). **(C)** Average (over neurons) standard deviation in time of the total input current in the two networks as a function of the input rate. **(D)** Average membrane potential in the two networks as a function of the external input rate. For reference, the panel shows also threshold potential (cyan), reset potential (green) and leak membrane potential (black). **(E)** Ratio of the decrease of the average MP observed in the two networks when increasing the external inputs as a function of the effective membrane time constant (see Figure 3A). The decrease in MP is computed for external inputs greater than 2 (spikes/ms)/cell with respect to the average MP obtained with an external input of 2 (spikes/ms)/cell. **(F)** Average (across neurons) standard deviation over time of the membrane potential in the two networks as a function of the input rate. Shaded area for COBN is not visible because it is too small. Results show that for the COBN both average total input current and membrane potential are almost constant across stimuli, while in the CUBN both quantities change dramatically for different input strengths. Cross-neuron variability of both variables is much higher in the CUBN. In both networks net input current fluctuations become larger when input rate is increased. This is reflected in larger fluctuations in the membrane potential in the CUBN, but not in the COBN. In panels **(A,B,D,E)** the average values of MP and input currents are computed over time and neurons.

**Figure 6:**
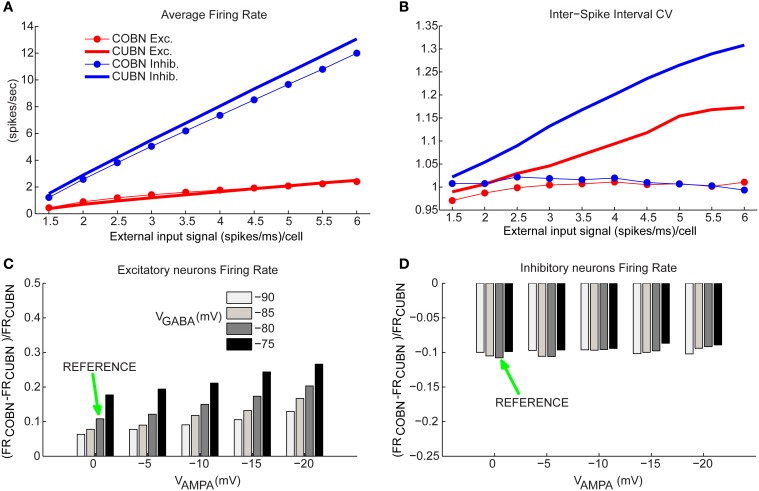
**Firing rates comparison. (A)** Comparison between average firing rate (FR) of inhibitory (blue) and excitatory neurons (red) for COBN (thin lines with markers) and CUBN (thick lines) as a function of the external input rate. **(B)** Average Coefficient of Variation of the Inter-Spike Interval in the two networks. Same color code as **(A)**. **(C)** Relative difference between the average FR of excitatory neurons in COBN and CUBN computed for different AMPA and GABA reversal potentials. The relative difference is averaged over the whole stimuli set ranging from 1.5 to 6 (spikes/ms)/cell. Green arrow indicates reference value of reversal potentials that were used in all the analysis (see Table 2). **(D)** Same as **(C)** for inhibitory neurons. In **(A,C,D)** the results are obtained from 50 trials of 4.5 s per stimulus, while for the panel **(B)** we used a single trial of 100.5 s per stimulus (see Methods). Results show that the two models have similar firing rates over the whole input range. This agreement is stable over a wide range of network parameters. On the other hand, the CV of the ISI increases with the input rate in the CUBN, while it does not in the COBN.

**Figure 7:**
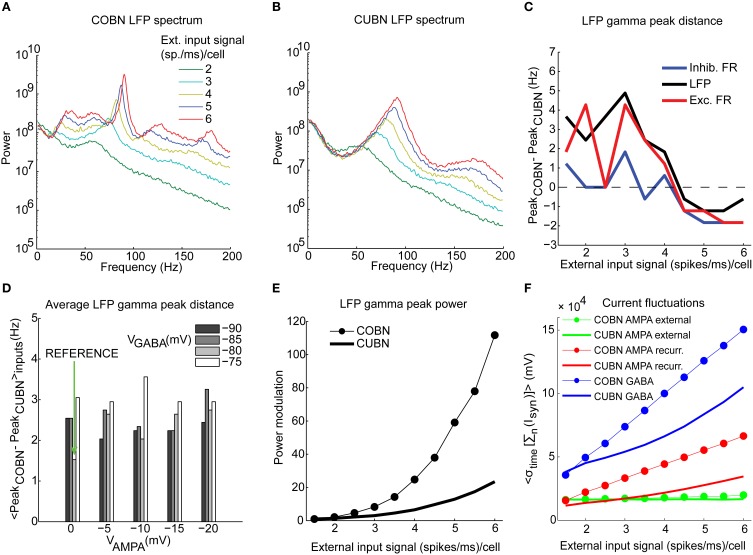
**Spectral dynamics of LFP and firing rate**. Input rate-dependent modulations of the LFP, studied focusing on position and amplitude of the gamma frequency peak. **(A)** LFP power spectra in COBN as a function of the external input rate. Data are averaged over trials. **(B)** Same as **(A)** for CUBN. **(C)** Difference in the position of the gamma band [(30–100 Hz)] peak of the power between the two networks. The analysis was performed for the LFP signal (black), and for the total firing rate of excitatory (red) and inhibitory neurons (blue). **(D)** Difference in the position of the LFP gamma peak averaged over the constant external inputs used (ranging from 1.5 to 6 (spikes/ms)/cell with steps of 0.5 (spikes/ms)/cell) as a function of AMPA and GABA reversal potentials. Green arrow indicates reference values (see Table 2). **(E)** Modulation of the LFP gamma peak power for the two networks. Power modulation is defined as the difference of the power of a frequency at a given input signal and its power at the input signal of 1.5 (spikes/ms)/cell, normalized to the latter power. **(F)** Average (over trials) amplitude of the fluctuations of the sum of the currents entering the excitatory neurons for the two networks as a function of the input rate. The currents are divided by the leak membrane conductance to obtain units of mV. Blue, red, and green lines represent GABA, recurrent AMPA and external AMPA respectively. These are the currents we used to compute LFP. Note that the external AMPA currents are almost identical between the two networks because their synapses are activated by the same spike trains in COBN and CUBN (see Methods). Results are computed by using 50 trials of 4.5 s per stimulus and show that (i) the gamma peak position across stimuli is similar for the two networks and this agreement is robust to change in the network parameters, (ii) the amplitude of the peak power is more modulated in the COBN because of the stronger fluctuations of the synaptic currents at the network level.

**Figure 8:**
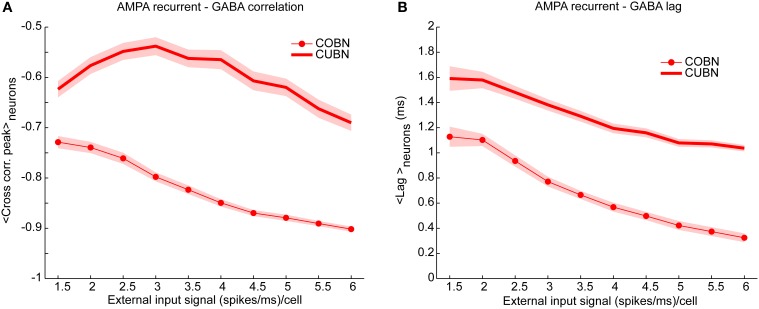
**Cross-correlation between AMPA and GABA inputs**. Cross-correlation between the time course of recurrent AMPA and GABA currents entering excitatory neuron. **(A)** Average peak value of cross-correlation between AMPA and GABA input currents into excitatory neurons (see Methods for details) for CUBN (thick line) and COBN (thin line with markers). Note that, AMPA and GABA currents having opposite sign, the correlation is negative. Shaded areas correspond to standard deviation across neurons. **(B)** Cross correlation average peak position. This measure quantify how much AMPA inputs lags behind GABA ones. Same color code as **(A)**. Results are computed by using a simulation of 10.5 s per stimulus and show that (i) correlation between recurrent AMPA and GABA input currents is stronger in the COBN than in the CUBN, (ii) input correlation decreases monotonously with input rate in COBN, while it does not in CUBN, (iii) GABA inputs lags behind AMPA inputs by few milliseconds in both networks.

**Figure 9:**
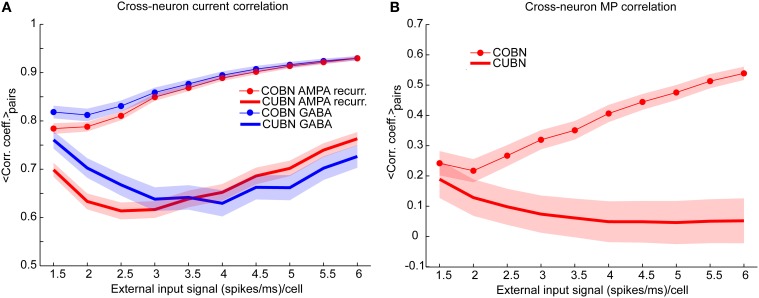
**Synaptic input and membrane potential correlation across neurons. (A)** Average cross-neuron correlation coefficient between the time course of recurrent AMPA currents (red lines) and GABA currents (blue lines) on excitatory neurons, for CUBN (thick lines) and COBN (thin line with markers), as a function of the external input rate. Similar results hold for inhibitory neurons (see “Rec. AMPA-Rec. AMPA” and “GABA-GABA” in Supplementary Table 1). **(B)** Average correlation coefficient between the membrane potential (MP) time courses of pairs of excitatory neurons as a function of the external input rate. While in the COBN the MP correlation increases with input rate, the opposite occurs in the CUBN. Shaded areas correspond to standard deviation across neuron pairs. Results are computed by using a simulation of 10.5 s per stimulus and show that in COBN the cross-neuron correlations between membrane potentials and between input currents are stronger than in CUBN.

**Figure 10:**
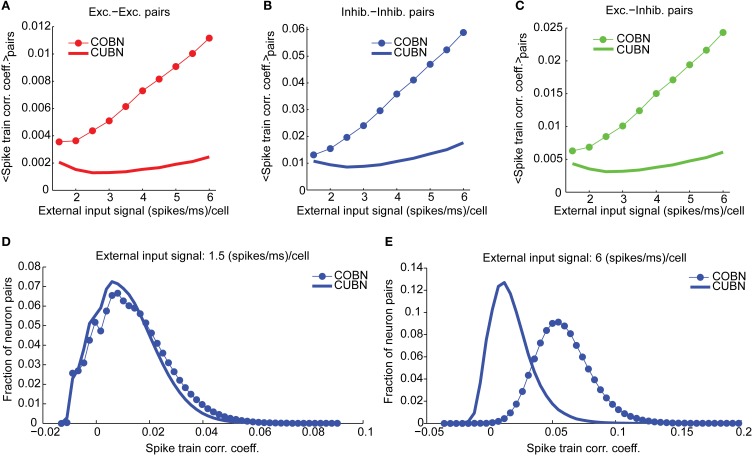
**Spike train correlation**. Spike train pairwise coefficient of correlation between neurons belonging to the same **(A,B)** or to different **(C)** populations. **(A)** Average spike train correlation between pairs of excitatory neurons as a function of the external input rate for CUBN (thick line) and COBN (thin line with markers). **(B)** Same as **(A)** for correlation between pairs of inhibitory neurons. **(C)** Same as **(A)** for correlations between pairs composed by an inhibitory and an excitatory neuron. **(D)** Distribution of the correlation coefficient across inhibitory neurons pairs for an input of 1.5 (spikes/ms)/cell for the two networks. **(E)** Same as **(D)** for an input of 6 (spikes/ms)/cell. Note that panels **(A–C)** do not have error bars for clarity, but the range of correlation values is similar to the one displayed in panels **(D,E)**. Results are computed by using a simulation of 100.5 s per stimulus and show that firing rate correlation is very low for both networks, and it increases with input rate in the COBN, but not in the CUBN.

**Figure 11:**
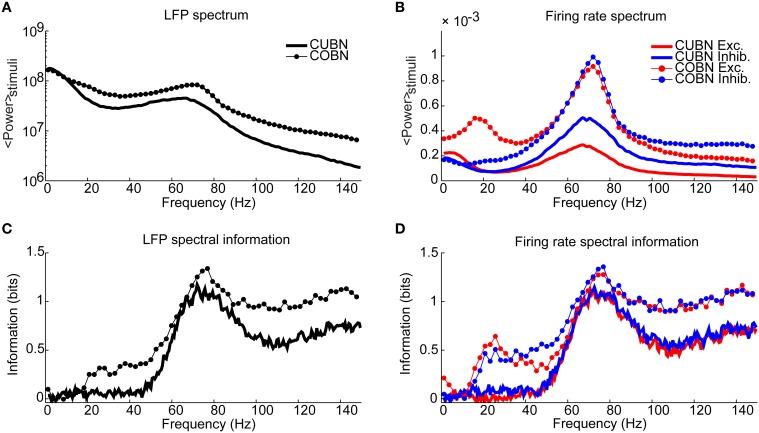
**Spectral information relative to the input rate**. Information carried by LFP power spectrum (left column) and population firing rates power spectra (right column) about constant inputs ranging from 1.5 to 3 (spikes/ms)/cell with steps of 0.1 (spikes/ms)/cell. Data are obtained by using 50 trials of 4.5 s per stimulus. **(A)** Average power spectrum of LFP over the entire stimulus range for the COBN and the CUBN (thin line with markers and tick line respectively). **(B)** Average power spectrum of the total firing rate of excitatory and inhibitory neurons (red and blue respectively) for the two networks [same line code as **(A)**]. **(C)** Spectral information carried by LFP about the input rate (see Methods for details). Same color code as **(A)**. **(D)** Spectral information carried by total excitatory and inhibitory firing rate about the input rate. Same color code as **(B)**. Results show that the COBN carries more information about constant stimuli for all considered frequencies, both in LFP and in firing rates.

**Figure 12:**
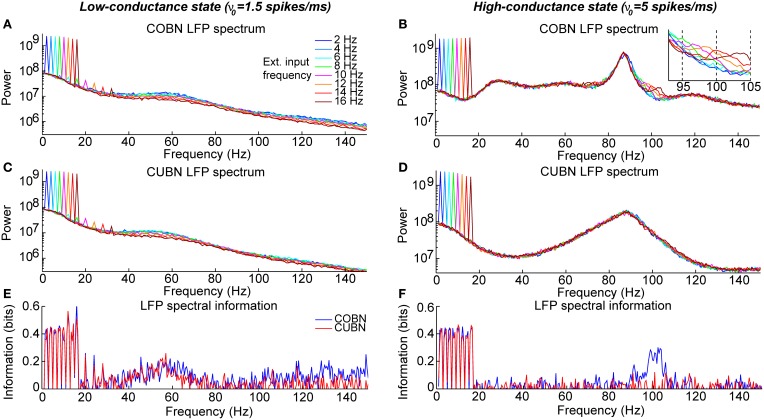
**Spectral information relative to periodic low frequency inputs**. Dynamics of the COBN and CUBN when injected with slowly oscillating inputs. The input signals are sine curves with amplitude A = 0.6 spikes/ms and frequency f, from 2 to 16 Hz, superimposed to a baseline of ν_0_ = 1.5 spikes/ms in the left column and ν_0_ = 5 spikes/ms in the right column. The first baseline value produces a low-conductance state, while the second originates a high-conductance state. Data are obtained from 50 trials of 10.5 s per stimulus. **(A,B)** LFP power spectrum in the COBN as a function of the external signal frequency. The power spectrum is averaged over trials. **(B)** Same color code as in **(A)**. **(C,D)** Same as **(A,B)** for the CUBN. The inset in **(B)** shows a detail of the panel in the frequency range where beats are displayed. **(E,F)** Spectral information carried by the LFP about the frequency of the stimulus presented (see Methods for details) for COBN (blue line) and CUBN (red line). Results show that the information due to the entrainment of the LFP to the slow input oscillations is almost the same in COBN and CUBN. The only difference is due to the beats that appear in the high-conductance state of the COBN [inset in **(B)**], which result in a peak of information around 100 Hz **(F)**.

**Figure 13:**
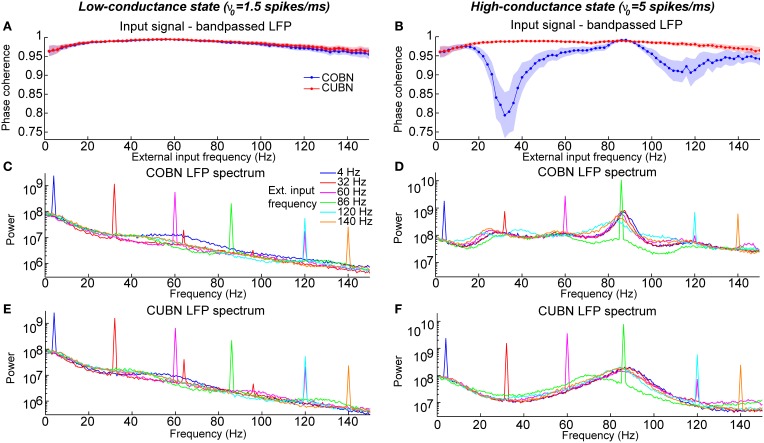
**Entrainment of LFP to input oscillations**. Entrainment of the network oscillations to the frequencies of the periodic input in COBN and CUBN. The input signals are periodic curves as in Figure 12, but with frequency *f* from 2 to 150 Hz. **(A,B)** Average (over trials) coherence between the phase of the input signal, with frequency *f*, and the phase of the LFP bandpassed in the corresponding frequency range (*f* − 1, *f* + 1) Hz (see Methods for details). Note that the phase coherence lies in the interval (0, 1). Data are obtained from 50 trials of 10.5 s per stimulus; shaded areas represent standard deviations across trials. Blue lines display results from COBN and red lines from CUBN. **(C,D)** LFP power spectrum in the COBN as a function of some selected external signal frequencies. The power spectrum is averaged over 50 trials. **(D)** Same color code as in **(C)**. **(E,F)** Same as **(C,D)** for the CUBN. In the low-conductance state both networks entrain very well to the external stimulus, whereas in the high-conductance regime the COBN entrains less well than the CUBN in the middle and in the highest frequency regimes.

**Figure 14:**
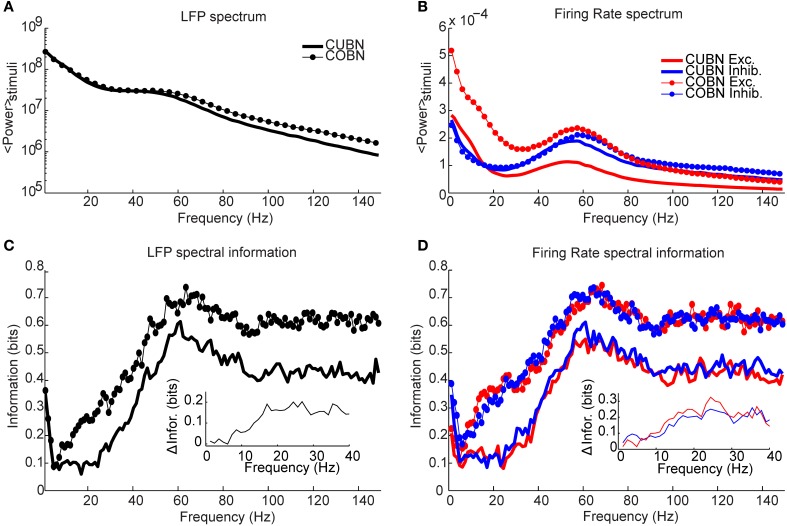
**Spectral information relative to naturalistic stimuli**. Information carried by LFP power spectrum (left column) and population firing rates power spectra (right column) about intervals of naturalistic stimulation based on LGN recordings in monkeys watching a movie. Recording time (80 s) is divided into 40 intervals, considered as different stimuli and the information is computed over 50 trials (see Methods for details). **(A)** Average power spectrum of LFP over the entire naturalistic input for COBN and CUBN (thin line with markers and thick line respectively). **(B)** Average power spectrum for the total firing rate of excitatory and inhibitory neurons (red and blue respectively) for the two networks. Same line code as in **(A)**. **(C)** Spectral information carried by LFP (see Methods for details). Same color code as in **(A)**. In the inset, it is shown the difference between COBN and CUBN information in the low frequency band. **(D)** Spectral information carried by total excitatory and inhibitory firing rates. Same color code as **(B)**. In the inset, it is shown the difference between COBN and CUBN information in the low frequency band. Results show that, also considering complex stimuli, the information relative to the mean value of the input [that here is the information carried by the frequencies above the delta band, (1–4) Hz] is higher and carried on a broader range of frequencies in the COBN, both in LFP and in firing rates. The information conveyed by delta band frequencies is instead almost identical in the two networks.

As explained above, we used three kinds of external input signals: constant input (Figures 2–11), periodic input (Figures 12, 13) and a naturalistic input (Figure 14). In the constant input case, each input rate, ν_0_, was considered a different stimulus (with simulations lasting 25.5 s), while, for the periodic stimuli, each stimulus corresponds to a frequency *f* (with simulations lasting 10.5 s). In the naturalistic case, the stimulus presentation time (80 s) was divided into 2 s long non-overlapping windows and each window was considered as a different “stimulus” for the information calculation, following the procedure described in (Belitski et al., 2008). We discarded an interval at the beginning of the simulations (500 ms both for constant and periodic case and 2 s for the naturalistic case) to avoid artifacts due to initial conditions. When computing information we considered three different response sets *R*: the average network firing rate, the average cross-neuron spike train correlation, and the LFP power of each single frequency (Belitski et al., 2008) in the (1–150) Hz range. To facilitate the sampling of response probabilities, the whole range of response values was divided into six consecutive intervals. Each of these intervals contained the same number of responses (i.e., they were equi-populated). All the responses belonging to a given interval assumed then the same interval-specific discrete value. In summary, we discretized the responses into six equi-populated bins. Then conditional probabilities *P*(*r|s*) were evaluated empirically by using the results from 50 trials per each stimulus *s*. We corrected information estimations for the limited sampling bias (Panzeri et al., 2007) by using the “quadratic extrapolation procedure” described in Strong et al. (1998) implemented in the Information Breakdown Toolbox (Magri et al., 2009).

## Results

We investigated the differences in the dynamics of neural populations between conductance-based LIF networks (COBNs) and current-based LIF networks (CUBNs), with particular emphasis in understanding how the neural population activity of these two types of network is modulated by external inputs. We first introduced an iterative procedure to determine synaptic parameter values so that the CUBN and the COBN were placed on a fair common ground, and could therefore be legitimately compared. We then analyzed similarities and differences of single neuron dynamics and of interactions among neurons in the two networks as a function of strength and nature of the external stimuli.

### Determining synaptic parameter values to build comparable current- and conductance-based networks

A necessary requirement to compare the activity of two different network models is to define a meaningful and sound correspondence between them. Our first step was thus to define a procedure to achieve comparable networks (see Methods for details). In brief, we set all the common parameters to exactly equal—and biologically plausible—values in both models. In this way the two models differed only because of the different synaptic model adopted: voltage-independent for CUBN (see Equation 5) and voltage-dependent for COBN (see Equation 6). In particular, the expression of the Post-Synaptic Currents (PSCs) in the COBN depended on conductances *g*_syn_ and on reversal potentials (*V*_AMPA_ and *V*_GABA_), while in the CUBN the PSCs depended only on synaptic efficacies *J*_syn_. We set *V*_AMPA_ and *V*_GABA_ at 0 and −80 mV respectively (but importantly our results were robust to changes in these parameters, see Figures 6C,D, 7D). We then used an iterative algorithm (detailed in Methods and illustrated in Figure 1) to set the values of the conductances *g*_syn_ of the COBN in such a way to obtain a COBN comparable to the CUBN with the given synaptic efficacies *J*_syn_.

The PSCs and the Post-Synaptic Potentials (PSPs) of recurrent AMPA and GABA synapses in the comparable networks are shown in Figures 2A,B,D,E for three different cases: current-based synapse, conductance-based synapse of a single neuron without background synaptic activity and conductance-based synapse of neurons embedded in the COBN network (that thus received background synaptic activity). The post-synaptic kinetics of conductance-based neurons is activity dependent. The terms that mediate this dependency are: the driving force (see Equation 6) and the increase of the total effective membrane conductance (see Equation 8). Both these terms tend to reduce the post-synaptic stimulus, but the PSCs are affected only by the driving force, while the PSPs by both the driving force and the effective membrane conductance. To understand how these two terms shape the post-synaptic stimulus, it is important to compare post-synaptic responses of conductance-based neurons, with and without background activity. Firstly, we compared PSCs and PSPs of the current-based synapse with those of the conductance-based synapse in the absence of background activity. In this condition the shape of excitatory PSCs and PSPs was almost identical for the two models when considering AMPA synapses (Figures 2A,D), while, for GABA synapses, differences between the two models were visible (Figures 2B,E). This asymmetry was due to the fact that the value of the average MP (see figure caption) was much closer to the reversal potential of GABA synapses than to the one of AMPA synapses (see Equation 12). Consequently the relative reduction of driving force during the post-synaptic event was higher for GABA synapses, provoking a stronger reduction of both PSCs and PSPs, with respect to the AMPA synapses (Figures 2B,E). Moreover, the PSPs of fast synapses (that is synapses with short τ_decay_) are less affected by synaptic bombardment (Koch, 1999; Kuhn et al., 2004), so, being the AMPA τ_decay_ shorter than the GABA ones (see Table 3), the asymmetry was even stronger when looking at the PSPs (Figures 2D,E). Secondly, we considered the conductance-based neurons embedded in the COBN and we found that in this case both AMPA and GABA synapses displayed a reduction in the amplitude and in the timescale, because the background network activity affected the time course of the MP (thus of the driving force) and increased the total effective membrane conductance.

As stated above, differences between the two synaptic models were expected to increase with input strength because the background synaptic activity increases. We measured this effect by injecting in the network constant inputs ranging from 1.5 to 6 (spikes/ms)/cell. Figures 2C,F show the amplitude of the different PCSs and PSPs as a function of the external input rate. Note that the PSCs (Figure 2C) and PSPs (Figure 2F) in the CUBN were activity-independent by construction, while, in the COBN, both PSCs and PSPs decreased substantially when input rate was increased; furthermore the relative reduction was the strongest for the slowest PSPs of GABA synapses (as stated above). Supplementary Table 1 reports average PSP amplitude values on both inhibitory and excitatory neurons.

Figure 2 shows that, in the COBN, PSPs were not only smaller but also faster than in the CUBN, consistently with previous results (Kuhn et al., 2004; Meffin et al., 2004). This reflected the decrease of the effective membrane time constant, τ_eff_, of the COBN, whose average value is shown in Figure 3A as a function of the input rate. When injecting stimuli with high input rates, we found that for both neuron populations the effective time constant, τ_eff_, was in the 1–5 ms range, matching experimental observations relative to the high-conductance states (Destexhe et al., 2003).

We then asked how the effective conductances associated with the AMPA and GABA currents varied in the COBN as a function of the input rate. We found (Figure 3B) that the average conductances grew linearly with input rate, as observed in single neuron case (Kuhn et al., 2004). Crucially, for high input rates, the relative conductances *g*_AMPA_/*g*_leak_ and *g*_GABA_/*g*_leak_ displayed values respectively close to 1 and 3.5, in the range of those found experimentally in high-conductance states (Destexhe et al., 2003). This suggested that our input range was suited to investigate the whole continuum going from low- to high-conductance states.

### Average single neuron properties

After having examined the properties of PSPs and conductances in the two comparable networks, we began investigating how these properties affect the dynamics of neural activity in the networks. To gain some visual intuition about this, we plotted (Figure 4) example traces of how variables reflecting single neuron and network activity evolve over time for the two types of network both in the low- and high-conductance state. The overall spike rate of individual neurons was similar for the two networks in both low- and high-conductance state (compare Figures 4A with 4C and Figures 4B with 4D) suggesting that the level of network firing was only mildly dependent on the synaptic model adopted. On the other hand, single neuron MP traces were similar in the two networks in the low-conductance regime (compare Figures 4E with 4G), but different in many aspects in the high-conductance regime (compare Figures 4F with 4H). In particular, in the high-conductance state, the COBN MPs had rapid gamma-range variations which were correlated across neurons and whose amplitude was more prominent than that of the gamma oscillations in the CUBN MPs, suggesting that the oscillation regime in the high-conductance state was tighter in the COBN than in the CUBN. Finally, we considered the traces of the LFP (which can potentially capture both supra- and sub-threshold massed neural dynamics). LFP traces were relatively similar across networks in the low-conductance state (Figure 4I). However, there was an interesting qualitative difference in the LFP traces in the high-conductance state: the COBN LFP had transient peaks of very high amplitude, which were not observed in the CUBN. At fixed level of overall firing rate, the amplitude of the LFP is modulated by the relative timing of the synaptic events contributing to it. Therefore this observation suggests that the COBN may undergo larger fluctuations in synchronization than the CUBN. The visual inspection of example traces suggests that, while some network properties such as overall firing rate are consistently close in the two networks, other more subtle aspects of network dynamics (such as the ability of the network to transiently synchronize its activity) may not be entirely equivalent in the two networks, especially in the high-conductance state. In the following we will systematically quantify this intuition.

An important feature of the models is the dynamics of the average (over time and neurons) of the total synaptic input current *I*_tot_ (Equation 4). We observed in both networks (Figure 5A) an increase of 〈*I*_tot_〉 with the input rate (Pearson correlation test, *p* < 10^−5^). However, 〈*I*_tot_〉 was significantly higher for the CUBN over all inspected inputs (*t*-test *p* << 10^−10^). The net input current 〈*I*_tot_〉 was also less modulated by the input rate in the COBN: the difference between the current (divided by the leak membrane conductance) at maximum and minimum input was 1 mV for COBN and 15 mV for CUBN. Even if the firing rate was very similar in the two networks (see Figure 6A), average GABA currents were weaker in COBN, while average AMPA currents were very similar (see Figure 5B). This discrepancy in the dynamics of the net input current was due to the fact that individual PSCs of GABA currents were more affected (i.e., reduced) by the change from CUBN to COBN with respect to the AMPA PSCs, as pointed out in Figure 2. Note also that in the case of external AMPA current, the spike trains that activated the synapses (more precisely the function *s*(*t*) in Equations 5 and 6) are exactly the same in the two models, while they were different for the other currents.

Consistent with the sample traces shown in Figures 4G,H, the average MP of the CUBN decreased steeply when we increased the input (−15 mV between maximum and minimum input, Figure 5D). This is due to the fact that, in the CUBN, the net input current strongly increased when increasing the external inputs (Figure 5A). Conversely, and consistently with the sample traces in Figures 4E,F, the decrease in COBN MP was smaller (−2 mV between maximum and minimum input, Figure 5D), consistent with previous results (Meffin et al., 2004). It is important to note that an increase of the input current led to an increase the voltage fluctuations in both models. However in the COBN, it caused also a concomitant increase of the membrane conductance, which in turn decreased the membrane voltage fluctuations. The dynamics of MP in COBN thus resulted from the competition between these two effects, which overall produced a suppression of both fluctuations and mean of the MP (Kuhn et al., 2004; Meffin et al., 2004; Richardson, 2004). We found that, for external inputs higher than 2 (spikes/ms)/cell, there was a linear relation (*R*^2^ = 0.98, *p* << 10^−10^) between the ratio of the average MP changes induced by the external inputs in the two networks and the effective membrane time constant of the COBN (see Figure 5E). This result confirmed and extended what found for a single neuron model in a high-conductance state in Richardson (2004). Shaded areas in Figures 5A,D indicate standard deviation across neurons, and show that the cross-neuron variability in both net input currents and MP was much larger in the CUBN than in the COBN, suggesting a more coherent activity for the latter (see subsection “Correlations among neurons**”**).

When we looked at the variability over time of the input currents, we found that it grew almost linearly and with very similar values for both COBN and CUBN (Figure 5C), while the increase of the variability over time of the MP was much more pronounced in the CUBN than in the COBN (Figure 5F). This result is still consistent with the suppression of voltage fluctuations typical of conductance-based model with respect to the current-based one.

In sum, our findings so far confirmed that dynamics previously observed in simpler conditions were valid also over a more extended range of conditions, proved that the range of input rates considered encompassed both low- and high-conductance regimes, and highlighted some of the differences between the dynamics of COBNs and CUBNs.

### Firing rate modulations

Having established a procedure that computes comparable CUBN and COBN parameters, and having investigated the synaptic responses in these comparable networks, we next compared the average firing rates of single neurons in the two networks, and studied how they are modulated by the strength of the input to the networks.

We considered individually the excitatory and inhibitory neural populations since they fired at very different rates (Brunel and Wang, 2003). Figure 6A shows the way inhibitory and excitatory firing rates increase with the input rate in the two networks. Consistently with the qualitatively intuition gained form the visual inspection of the raster plots in Figures 4A–D, we found that the discrepancies between COBN and CUBN firing rates were extremely small (average difference over external inputs of 10%), though significant (*t*-test *p* < 0.05 except for excitatory neurons with external input rates greater than 4 spikes/ms). This shows that the algorithm used to set comparable networks produces networks whose neurons have similar average firing rates with a similar dependence on the input strength, both in low- and high-conductance states.

To verify if the agreement of the firing rate in the two comparable networks was robustly achieved over a wide range of parameters, we computed the COBN synaptic conductances for a set of 20 different COBN networks (obtained by using the setting procedure illustrated in Figure 1 with 20 different combinations of the synaptic reversal potentials, *V*_AMPA_, ranging from 0 to −20 mV, and *V*_GABA_, ranging from −75 to −90 mV). We then computed the average firing rates for each resulting network. We found that even when *V*_AMPA_ was −20 mV and *V*_GABA_ −75 mV, and hence the discrepancies between the two models were stronger, the excitatory neurons firing rate differed between COBN and CUBN at most by 25%, but usually the difference was much smaller, on the order of 10% (Figure 6C). Note that, given the very low firing rate of excitatory neurons, the relative difference corresponded always to small values of absolute difference (<0.4 spikes/ms). The difference in the firing rate of the inhibitory neurons between COBN and CUBN were of the order of 10% for all reversal potentials combinations inspected (Figure 6D).

These results show that our procedure determines COBNs with firing rates similar to the compared CUBN for a wide range of parameters. In current-based neurons the firing rate is modulated only by the increase in the MP fluctuations (Figure 5F), while in conductance-based neurons, the firing rate activity is the result of two different competing effects: the shortening of the timescales (Figure 3A) and the increase of the membrane fluctuations (Figure 5F), that tend to facilitate the firing activity, and the increase of the effective membrane conductance, that acts in the opposite direction (Figure 3B) (Kuhn et al., 2004; Meffin et al., 2004; Richardson, 2004). It is therefore quite interesting that these underlying different dynamics compensate to produce, in the two corresponding network models, very similar firing rates over a wide range of inputs and parameters.

We then considered how the coefficient of variation (CV) of the inter-spike interval (ISI) changed with the strength of the input rate. We found (Figure 6B) that the two networks showed a very different dependence of CV on input rates. The ISI CV of neurons of the COBN was close to one for all considered input rates (indicating near-Poisson firing statistics). In contrast, in CUBN, the ISI CV was higher than 1 (i.e., the firing was more variable than that of a Poisson process) and increased with the input rate, reaching values up to 1.33 and 1.16 for inhibitory neurons and excitatory neurons respectively, confirming results of (Meffin et al., 2004). The difference between the CVs of neurons in COBN and CUBN was highly significant (*t*-test, *p* < 10^−7^) for all input rates above 1.5 spikes/ms. The larger ISI CV of neurons in COBN was consistent with our finding of larger MP fluctuations in time in the COBN (Figure 5F). ISI CV values were within the experimentally observed range 0.5–1.5 (Maimon and Assad, 2009) for both networks, but only the COBN reproduced the experimental result that the ISI CV of cortical neurons is not affected by the firing rate (Maimon and Assad, 2009).

The discrepancy between the similarity of the firing rates and the dissimilarity of the ISI CVs suggests that the first-order statistics of the two networks were close to match, but the second order statistics differed significantly.

### Spectral modulations in simulated local field potentials

We investigated then the differences in the spectral modulations of network activity, as measured by the simulated LFP and by the total excitatory and inhibitory firing rate generated by the two networks. LFP models can offer interesting insights into the dynamics of cortical networks (Einevoll et al., 2013) because they offer an insight in both supra- and sub-threshold dynamics that can be compared with experimental recordings; however the differences in LFPs computed from networks with either current- or conductance-based synapses have not been investigated yet. We expected significant differences to arise because, as detailed above, the sub-threshold dynamics of COBNs and CUBNs were quite different.

The dependence of LFP spectrum on the input rate (Figures 7A,B) shows that, consistent with previous results (Brunel and Wang, 2003; Mazzoni et al., 2008, 2011), both networks develops gamma range (30–100 Hz) oscillations that become stronger and faster as the input is increased. Figures 4I,J illustrate this effect in the time domain. Figures 7A,B show the LFP input rate-driven modulation in COBN and CUBN. The dependence of response to variations in input rate in the two networks was qualitatively similar. There was no modulation for frequencies below 5 Hz (Pearson correlation test, *p* > 0.1); there was strong modulation in the gamma band and above (Pearson correlation test, *p* < 0.01). The difference between the position of the COBN and CUBN gamma peak was always below 5 Hz (Figure 7C). For comparison, we also computed the power spectrum of the total firing rate of excitatory or inhibitory neurons (Figure 7C). The spectral peaks of COBN and CUBN were very close also in this case.

We tested the robustness of the agreement between spectral peaks of CUBNs and COBNs by measuring the average (over stimuli) gamma-peak distance between the two networks for different AMPA and GABA reversal potentials (similarly to what was done in the analysis represented in Figures 6C,D), and we found that the two networks always displayed almost identical positions of the gamma frequency peaks (Figure 7D).

Note that we did not build the comparable networks to obtain robustly similar firing rates and similar dominant frequencies in the gamma band, as we used other constraints to select comparable parameters. The equivalence and robustness of rates and gamma peaks arose from network dynamics, and, in particular, the robustness corroborates the notion that our procedure indeed produces a meaningful comparison. We also tested other kinds of procedures to set the COBN synaptic conductances, *g*_syn_, given the CUBN synaptic efficacies, *J*_syn_. In particular we define *g*_syn_ such in a way to maximize the similarity of PSCs (in one case) or PSPs (in another case) between the two networks at the single neuron level, to compensate for the post-synaptic stimulus reduction that is peculiar of the COBN with respect to the CUBN (Figure 2). When using these procedures the results were both less robust to change in the synaptic reversal potentials and less similar between CUBN and COBN (data not shown).

On the other hand, differences between the LFP spectra of the two networks are also apparent in Figures 7A,B. First, the COBN gamma peak was larger and was modulated by the input rate in a much stronger way than the CUBN gamma peak (Figure 7E). Given the fact that the net input current in the COBN was smaller (Figure 5A) and also fluctuated slightly less than in CUBN (Figure 5C), at first we found this result surprising. However, the phenomenon can be understood after measuring the AMPA and GABA fluctuations. As reported in Figure 7F, the size of recurrent AMPA and GABA current fluctuations was larger in COBN than in CUBN, and the difference increased with the input rate. Indeed, while the simultaneous increases of AMPA and GABA fluctuations compensated each other in the COBN net input current (Figures 5A,B), the contributions of these two currents to the computed LFP have the same sign (see Methods), and this led to a stronger spectral peak in the COBN. Second, the CUBN displayed a broad LFP spectral peak in the high gamma region (>60 Hz), and small fluctuations in the low gamma region (<60 Hz), while, in the COBN, for inputs greater than 3 (spikes/ms)/cell there was a sharp peak in the high gamma band and also a pronounced plateau in the low gamma. Third, since the power associated with this plateau was modulated by the input rate, for the COBN all frequencies above 20 Hz were significantly modulated, while in the CUBN significant modulation occurred only for frequencies above 60 Hz. As we will see in the next section, the narrower gamma peak indicates a stronger synchronization in the COBN than in the CUBN, while the stronger modulation in the gamma power makes the amount of information conveyed by the COBN larger than in the CUBN (see “Information about external inputs” subsection).

For both networks, the spectra of the total firing rate were qualitatively very similar to the spectra of the LFP for all input rates considered (data not shown). Therefore all the aforementioned differences were present also when comparing the COBN and CUBN total firing rate power spectra.

### Correlation between AMPA and GABA currents

The correlation between AMPA and GABA synaptic currents is known to play a very important role in determining the network dynamics and in particular the spike train variability (Isaacson and Scanziani, 2011). A negative correlation of AMPA and GABA input currents leads to sparse and uncorrelated firing events, while positive values lead to strong bursty synchronized events (Renart et al., 2010). We thus compared the cross correlation between recurrent AMPA and GABA currents impinging on single neurons in COBN and CUBN. We found that the correlation between GABA and AMPA inputs was stronger (i.e., more negative) in the COBN for all external input rates (Figure 8A). Moreover, in both networks, AMPA currents led GABA currents with lags shorter than 5 ms, of the order of those observed in (Okun and Lampl, 2008). However, for all external input rates, AMPA-GABA lags were smaller in the COBN (Figure 8B). Although Figure 8 shows results only for excitatory neurons, similar results held for inhibitory neurons (Supplementary Figure 2). Finally, these results held also when using as external noise a white noise process instead of an Ornstein-Uhlenbeck process (see Supplementary Figure 4C).

### Cross-neuron correlations

The fact that the cross-neuron variability in average current inputs and MPs was much smaller (Figures 5A,D) and high gamma frequency peaks were narrower in the COBN (Figures 7A,B) suggested that the activity was more coherent in the COBN than in the CUBN. This view was further corroborated by the finding that the sum of the recurrent currents was larger in the COBN (Figure 7F) and suggested that, in this network, input currents may be more correlated across different neurons.

We verified this hypothesis by measuring the average Pearson correlation coefficient between the time evolution of the recurrent AMPA and of the GABA input currents over neuron pairs (see Methods), Figure 9A shows that for both AMPA and GABA currents the average cross-neuron correlation coefficient was indeed significantly stronger (*t*-test, *p* << 10^−10^) in the COBN for all external input rates. Figure 9A shows also that, in the COBN, the cross-neuron correlation grew with the external input rate for both currents (Pearson correlation test, *p* < 10^−5^). In the CUBN the AMPA currents were linearly correlated to the input rate (Pearson correlation test, *p* < 0.05), while GABA currents varied with the input rate in a non-monotonic way. However, if we used white noise, instead of the Ornstein-Uhlenbeck noise (see Methods), the cross-neuron current correlation was again higher in the COBN (*t*-test, *p* << 10^−10^), but grew monotonously with the input rate for both networks (Pearson correlation test, *p* < 10^−5^), as shown in Supplementary Figure 4A. The increase in the difference between the cross-neuron current correlation in COBN and CUBN with the input rate (Figure 9A) led to the increase of the difference in AMPA and GABA total fluctuations in the two networks, shown in Figure 7F. To fully appreciate the key role played by correlations note that, if the correlations were similar in COBN and CUBN, fluctuations would be expected to be larger in CUBN since the firing rate was similar for the two networks (Figure 6A) and the single PSC amplitude was larger for the CUBN (Figure 2). Cross-neuron correlation of the input currents should be reflected in cross-neuron MP correlation. The previously shown sample traces of the MP of neuron pairs (Figures 4E,H) suggested that the correlation was indeed similar for COBN and CUBN in the low-conductance state, but much stronger for the COBN in the high-conductance state. We thus analyzed the average correlation of the MP time courses of pairs of excitatory neurons (Figure 9B). Over the whole external input range considered, MP correlation in the COBN was significantly stronger than in the CUBN (*t*-test, *p* << 10^−10^). Cross-neuron MP correlation in the COBN increased with external input rate (Pearson correlation test, *p* < 10^−8^), while it was only mildly affected in the CUBN (Pearson correlation test, *p* < 0.02). These results held for all considered neuron pairs (Supplementary Figure 3) and also when considering white noise, instead of Ornstein-Uhlenbeck noise (Supplementary Figure 4B).

We finally computed the cross-neuron spike train correlation. We expected it to be related to the MP correlation displayed in Figure 9B, even if, since both networks were in a fluctuation-driven state, the spike train correlation should be close to zero (Brunel and Wang, 2003; Renart et al., 2010). We found indeed a very low average spike train correlation (Figures 10A–C) such that, for low input rates, a significant fraction of pairs displayed negative correlation (Figure 10D). However, in the CUBN, the spike train correlation was weaker and less sensitive to input rate changes than in the COBN (see Figures 10A–C and compare Figures 10D,E). These results did not change if we injected white noise, instead of Ornstein-Uhlenbeck noise, in the network (Supplementary Figure 4D).

### Information about external inputs

In the previous subsections we investigated how the average level of spike rate, LFP and spike train correlation depends on the external input to the network, finding a more pronounced stimulus modulation of LFP gamma power and of cross-neural correlation in COBN. To quantify these stimulus modulations of network activity, we computed the mutual information between the stimuli to the network and various aspects of network activity (see Methods for details).

We first measured the information carried by the average firing rate, both of excitatory and inhibitory neurons, in the two networks by using constant stimuli in the range 1.5–3 (spikes/ms)/cell with steps of 0.1 (spikes/ms)/cell. We found that, consistently with the results shown in Figure 6A, the information carried by the average firing rate had the same value of 2.3 bits for both neural populations in both network models. Given that the modulation of spike train correlation with external input is greater in the COBN than in the CUBN, we expected that also the mutual information between the spike train correlation and the input rate was greater in the COBN than in the CUBN. Indeed this was the case: information in spike train correlation was much larger in the COBN (1.6 and 2.0 bits for excitatory and inhibitory neurons respectively) than in the CUBN (1.4 and 0.9 bits for excitatory and inhibitory neurons respectively).

We measured then the information content of the LFP power spectrum. The LFP power spectrum averaged over all the presented constant stimuli was higher for the COBN than for the CUBN for all frequencies above 15 Hz (Figure 11A). We found that, at all frequencies above 20 Hz, the COBN LFP spectrum carried more information about input rate than the CUBN LFP spectrum (Figure 11C). Most notably, the peak information increased by about 20%, and the (20–45) Hz frequency range was informative in the COBN, but not in the CUBN. We repeated the analysis considering the power spectra of the total inhibitory and excitatory firing rate in the two networks. Excitatory neurons in the COBN had stronger power than excitatory neurons in the CUBN for all frequencies (Figure 11B, note that here the y-scale is linear, while in 11A is logarithmic) and showed a secondary peak at about 20 Hz. For inhibitory neurons, instead, the COBN power spectrum was higher only for frequencies above 15 Hz, as in the LFP.

So far we have investigated only the information carried about the strength of a time-independent input to the network. In a previous work on CUBN (Mazzoni et al., 2008) it has been shown that when the input to the CUBN is dominated by low frequency fluctuation, the network oscillations (captured by both LFP and massed firing rate measures) form two largely independent frequency information channels. A gamma-range information channel is generated by recurrent interactions of inhibitory and excitatory neurons and conveys information about the mean input rate. A low-frequency information channel is generated by entrainment of the low frequency network activity to the slow fluctuations of the input stimulus and carries information about the stimulus time course on such slow time scales. We wanted to test how these two information channels, developed when presenting the network with time-varying stimuli, depended on the choice of the synaptic model.

To investigate this point, we injected into the two networks periodic stimuli with fixed amplitude and frequency varying between 2 and 16 Hz. These input frequencies below 16 Hz were taken to represent the slow naturalistic fluctuations present in natural input signals (Luo and Poeppel, 2007; Chandrasekaran et al., 2010; Gross et al., 2013). Since we wanted to investigate potential differences between models separately in low- and high-conductance states, we generated two kinds of input signals: a low-input regime (corresponding to a low-conductance state) and a high-input regime (corresponding to a high-conductance state). Thus the periodic input was made of a sinusoidal signal at a given frequency superimposed to a constant baseline that was set to a low value (ν_0_ = 1.5 spikes/ms) to induce a low-conductance state and to a high value (ν_0_ = 5 spikes/ms) to induce a high-conductance state. The amplitude of the sinusoidal component of the input was 0.6 spikes/ms across all simulations. Results are reported in Figure 12.

We examined first the low-conductance state (left column of Figure 12). We considered the LFP power spectra of the two networks in response to periodic stimuli of different frequencies (Figures 12A,C). With respect to the previously examined constant input case (Figures 7A,B), the LFP power spectrum of both networks had an additional high narrow peak exactly at the same frequency of the periodic input. This peak signaled the entrainment of the network to the periodic input (Mazzoni et al., 2008). The ability of the two networks to entrain their dynamics to the low-frequency stimuli suggested that the power of the LFP at such low frequencies could discriminate which of these periodic inputs was being presented. We tested this suggestion quantitatively by using mutual information, and we found that the slow LFP frequencies conveyed indeed information about the stimuli, approximately in the same amount in both networks (Figure 12E). Note that, in the low-conductance state, there was also a slight modulation with the input frequencies of the power in the gamma band (40–70) Hz, with slightly lower gamma power for stimuli of faster frequency (Figures 12A,C). These modulations of gamma-range power resulted in moderate amounts of stimulus information in the same range, (40–70) Hz, (Figure 12E), and were likely due to the time taken by the networks to develop gamma oscillations following the very low input values occurring at the trough of the sinusoidal input.

We then investigated the high-conductance state (right column of Figure 12). Figures 12B,D shows that entrainment of both networks to low frequencies (signaled by the high narrow peak of LFP spectrum at the same frequency as the input) occurred strongly in the high-conductance state. The information about which of these periodic inputs was being presented, carried by the low frequency LFP power, was still identical in the two networks (Figure 12F). Moreover, and consistently with the above results obtained with constant inputs (Figures 7A,B), the gamma peak in the high-conductance states was much stronger and narrower in the COBN than in the CUBN. Probably because of this, the COBN (but not the CUBN) developed beats of the low-frequency peaks into the frequency range around 100 Hz (inset Figure 12B). Since the low-frequency peak varied with the input, these beats led to an amount of information in the COBN LFP power around 100 Hz. The moderate gamma-range information peak, observed in the (40–70) Hz range for the low-conductance state (Figure 12E), was absent in both networks for the high-conductance regime (Figure 12F), because the input rate was always high at any time point. Thus gamma oscillations in the range (80–94) Hz were always strong, with relatively small fluctuations over time, leading to not discernable modulation across the set of input frequencies considered (Figures 12B,D).

We then investigated the ability of the network to entrain to a wider range of input frequencies, in particular including frequencies as fast as or faster than the gamma oscillations intrinsically generated by the network. We did so by testing the network with periodic stimuli over the 2–150 Hz range of input frequencies (Figure 13). Again, to investigate differences between models separately in low- and high-conductance regimes, we generated two kinds of input signals that only differed for the value of the baseline, as described above. We quantified entrainment by computing the coherence between the phase of the input signal and the phase of the LFP bandpassed in a narrow band (with 2 Hz bandwidth) centered at the frequency of the periodic input. In the low-conductance state both networks were strongly entrained to the input over the whole range of frequencies examined, as indicated by the high phase coherence (Figure 13A). However, when injecting the same input frequencies with the highest baseline (i.e., making the network operate in a high-conductance state), the behavior of the two networks was very different. The CUBN could still entrain extremely well over the entire input frequency range tested. The COBN entrained extremely well to inputs in the (80–94) Hz input frequency range, but less well to inputs with frequency between 16 Hz and 80 Hz, and above 94 Hz. The reason for the presence in the COBN of frequency regions with lower phase coherence (and thus less accurate entrainment to the periodic input) may be because, in the high-conductance state, the COBN had stronger internally generated recurrent oscillations (of higher power than the CUBN, see Figures 13D,F) whose dynamics likely did not interfere constructively with the dynamics of the entrainment to the input. This resulted in peaks of less high amplitude in the COBN LFP spectrum at the exact frequency of the periodic input (Figures 13D,F). It is interesting to note that the COBN still entrained very well in the (80–94) Hz input frequency range (Figure 13B), despite this was also the frequency range exhibiting the strongest recurrent oscillations. Indeed, this range coincided with the peak amplitude of the internally generated gamma oscillations (Figure 12B). The ability of the network to entrain well in this gamma range can be understood by observing that this was also the range more strongly modulated by the input rate (Figure 7A). Thus, due to their particularly strong responsiveness to the input, external and internal oscillation in this range could interfere constructively, resulting in large peaks of the network LFP at the input frequency (Figure 13D).

To study the differences in the responses of the two networks to stimuli more complex and more biologically relevant than periodic functions, we finally compared the information carried by the LFP and firing rate spectra in COBN and CUBN when using the naturalistic time-varying inputs. We injected then in the networks naturalistic stimuli based on MUA recordings from the LGN of an anaesthetized macaque presented with a commercial 80 s color movie clip. The average LFP and total firing rate power spectra for both networks with this set of stimuli are displayed respectively in Figures 14A and B. All these spectra had higher power at low frequencies (as the input signal had), and the gamma peaks were low because the average stimulus rates were in the range 1.2–2 spikes/ms. We computed information about which part of the time-varying naturalistic signal was being presented (see Methods for details). We found that both LFP and firing rates spectra carried more information in the COBN than in the CUBN, for all frequencies (Figures 14C,D). The difference in spectral information between COBN and CUBN for frequencies below 5 Hz was almost zero for the LFP and very low for the firing rates (see insets of Figures 14C,D).

Our findings therefore confirm that the two independent information channels (one in the low frequencies due to the entrainment to the input, and one in the gamma band due to internally generated oscillations), which were previously reported for the CUBN (Mazzoni et al., 2008), also exist in the COBN. Moreover, our results show that the information about the input conveyed by low frequencies, both in low- and high-conductance states, does not depend on the details of the synaptic model adopted, while the information encoded in the gamma range is larger in the COBN than in the CUBN.

## Discussion

Here we compared in detail the neural population dynamics of LIF networks with either current-based or conductance-based neuron models. The comparison of network dynamics was made on networks with all shared parameters set to an equal common value, and with model-specific synaptic parameters set by a novel recursive procedure that makes COBN and CUBN directly comparable. Our main result was that, although average firing rates and peak frequency of gamma oscillations in such comparable networks were very similar over a wide range of parameters, other aspects of neural population dynamics (such as shape of oscillation spectra or cross-neuron correlation) were significantly different between CUBN and COBN. In particular, oscillation spectra, gamma synchronization and cross-neuron correlation were more markedly modulated by the external input in COBN than in CUBN. The significance of these findings, and their relationship with both theoretical and experimental literature, is discussed in the following.

### Establishing comparable networks

The first contribution of the work presented here was to provide a new recursive algorithm to determine the COBN conductance values that correspond to a given set of CUBN synaptic efficacies in networks that have identical values for all the shared parameters. We found that this procedure was able to build two networks displaying relatively small differences, both in the average firing rates and in the gamma frequency peak position, for an input range sufficiently large to encompass both low- and high-conductance states (Destexhe et al., 2003). The relationship of our new procedure with the previous work we built on is discussed in the following.

In a previous work addressing the issue of building equivalent CUBN and COBN models (La Camera et al., 2004), the authors discarded the approach of setting synaptic conductances at fixed average MP (i.e., the one we used in this work) stating that “Although this might work for a single input, it does not work for all inputs in a large pool (results not shown).” La Camera and colleagues proposed instead to build equivalent networks by making both inhibitory and excitatory connectivity free parameters, so that the optimal equivalence was obtained when the CUBN had twice the excitatory and half the inhibitory connectivity of the COBN. Differently from this procedure, in our work all the common parameters of the two networks were identical, including the connectivity matrix. This, in our view, has the advantage that differences in network dynamics can be more directly imputed to changes in model synaptic dynamics. Meffin et al. (2004) determined the value of the conductances starting from a “fixed rough estimate of the average MP” set as the midpoint between threshold and reset potential. The difference with our work is that we used directly the actual average value of the MP of the neurons of each population. Note that there is a discrepancy between the two values since the true average MP was equal or slightly below the reset potential (Figure 5D). In extensive initial simulations, we found that using the average MP, rather than the midpoint between threshold and reset potential, made it much easier for the comparable networks to exhibit very close firing rates and gamma spectral peaks (results not shown).

In summary, the comparable networks established with our procedure exhibited average firing rate and position of the peak of the LFP power spectrum that were both similar across network models and were relatively robust to changes in the synaptic reversal potentials. In our view this strengthens the value and usefulness of the setting procedure introduced.

### Comparison between synaptic models

Previous seminal papers (Kuhn et al., 2004; Meffin et al., 2004; Richardson, 2004) compared the firing rate and MP of conductance- and current-based LIF neurons. Our findings, summarized in Supplementary Table 1, confirmed the main results of these previous works, and extended them in several ways. Our main contribution was to extend the comparison to include other aspects of neural population dynamics. In particular, we considered the effect of the synaptic models on the spectrum of network activity, on the cross-neuron correlations and on the stimulus modulation of these different network features. The significance of these advances is discussed in more detail below.

### Correlation in the networks

Spike trains of different neurons were more correlated in the COBN than in the CUBN, with the correlation difference increasing with the external input rate. The fact that the COBN spike train correlation was more strongly modulated by the input rate led to the fact that spike train correlation carried more information in the COBN.

In our networks, the neurons received inputs from the same simulated external pool and this led to values of shared input that were likely higher than those shared by pairs of cortical neurons recorded from different electrodes. However, in the COBN, the dependence of correlation on the network stimuli resembled qualitatively the one observed in real experiments, more than in the CUBN. First, the positive correlation between firing rate intensity and spike train correlation is often observed in neurophysiological experiments, (Kohn and Smith, 2005), and this behavior is only reproduced by the COBN. Further, MP of cortical neurons (Lampl et al., 1999) (but see also Yu and Ferster, 2010) are more correlated when they receive an input triggering a stronger response (i.e., having an higher contrast/the correct orientation). This resembles the dynamics displayed here by the COBN, but not by the CUBN. Moreover, in several experiments (see Isaacson and Scanziani, 2011 and references therein), the correlation between AMPA and GABA synaptic inputs is stronger the more intense is the stimulus, consistent with the COBN dynamics shown in Figure 8A.

The high values of correlation that we found in the COBN might, at first sight, look different from those of Renart et al. (2010) in which a conductance-based LIF network, with a structure similar to the one considered here, displayed a much smaller MP correlation thanks to the decorrelation due to a precise balance between excitation and inhibition. In other words, in that work, AMPA-GABA correlation and cross-neuron MP correlation were described as mutually exclusive. We think that the reason for the difference between their results and those obtained in our work is the crucial assumption of Renart et al. (2010) that AMPA and GABA timescales are identical. In a supplemental analysis the authors showed indeed that, when AMPA synapses were made progressively faster than GABA, the negative feedback was not fast enough to compensate for excitation and hence to decorrelate the neurons; the network became then more synchronized. When in Renart et al. (2010) the authors considered the case in which τ_*rE*_ = 2 ms and τ_*rI*_ = 5 ms (very close to our values, see Table 3), the correlation between GABA and AMPA currents reached values above 0.5, coherent with our results (Figure 8A).

### Frequency spectra of network activity

We also compared the frequency spectra of the network activity in COBN and in CUBN. A marked difference was in the larger amount of information and stronger stimulus modulation of the gamma range for COBN. This, in our view, may be explained as follows. When increasing the external input rate, we observed an increase of the cross-neuron spike train correlation in the COBN, which was associated with an increase of the cross-neuron correlation of the synaptic currents (both AMPA and GABA). This caused a stronger modulation of the COBN currents and consequently of the LFP gamma peak. The stronger modulation of the gamma band in turn contributed to the fact that, both when time-constant and time-varying inputs were injected, the COBN carried more information than the CUBN in the gamma band.

Neurophysiological recordings of LFP spectra modulation in visual cortex during stimulation with various kinds of visual stimuli (Henrie and Shapley, 2005; Belitski et al., 2008) reported much broader gamma peaks than the ones we found for COBNs. The width of gamma peaks reported in cortical data was more similar to the broad gamma peak generated by CUBN rather than to the sharp peak generated by the COBN. We hypothesize that the sharpness of the COBN gamma peak may be over-emphasized by the lack of neuron-to-neuron heterogeneity in the specific network models implemented here. Introducing a small degree of variability in neuronal parameters could decrease the correlation in COBN while keeping it stimulus-dependent. An important point for future research is to understand how heterogeneities in network parameters differentially affect COBN and CUBN dynamics.

A final point worth discussing is that the COBN, unlike the CUBN, showed considerable amounts of information about input strength in the LFP power in the frequency range 15–25 Hz. Notably, the power of real visual cortical LFPs (Belitski et al., 2008) also did not carry information in this frequency range. Belitski and coworkers hypothesized that the 15–25 Hz LFP frequency region related mainly to stimulus-independent neuromodulation. The additive contribution to the LFP of fluctuations generated by a stimulus-unrelated system would potentially cancel out the information generated by the network in this frequency range.

### Conflict of interest statement

The authors declare that the research was conducted in the absence of any commercial or financial relationships that could be construed as a potential conflict of interest.
