# Supplementary material for: Comparison of the dynamics of neural interactions between current-based and conductance-based integrate-and-fire recurrent networks
Source: Front Neural Circuits. 2014 Mar 5;8:12. doi: 10.3389/fncir.2014.00012 (PMC3943173; doi:10.3389/fncir.2014.00012)
Supplement: Supplementary file 1 [file DataSheet1.ZIP › CavallariEtAl2014/LIF_COBN/ReadMe_COBN.pdf]

Recurrent random network with excitatory and inhibitory Leaky Integrate-and-Fire (LIF) neurons with conductance-based synapses from the paper “Comparison of the dynamics of neural interactions between current-based and conductance-based integrate-and-fire recurrent networks” written by S.Cavallari, S.Panzeri and A.Mazzoni and published in Frontiers in Neural Circuits (2014), 8:12. doi:10.3389/fncir.2014.00012. The paper compares the activity of this conductance-based network (i.e. code_COBN.c) with the activity of a comparable network of LIF neurons with current-based synapses (whose source code is in the “LIF_CUBN” folder).

The function code_COBN.c is a mex source code. You have to compile this routine in Matlab to generate the mex file, e.g.: code_COBN.mexw64

To compile the routine you have to include the functions ran1.c and gasdev.c in the compiling instruction in the Matlab workspace, in this way:

mex code_COBN.c ran1.c gasdev.c

After you compiled the function, you can call it as specified in the help. For more information use the help of the function:

help code_COBN

Question on how to use the model should be addressed to stefano.cavallari@iit.it
