# Supplementary material for: Comparison of the dynamics of neural interactions between current-based and conductance-based integrate-and-fire recurrent networks
Source: Front Neural Circuits. 2014 Mar 5;8:12. doi: 10.3389/fncir.2014.00012 (PMC3943173; doi:10.3389/fncir.2014.00012)
Supplement: Supplementary file 2 [file Presentation1.PDF]

Supplementary material

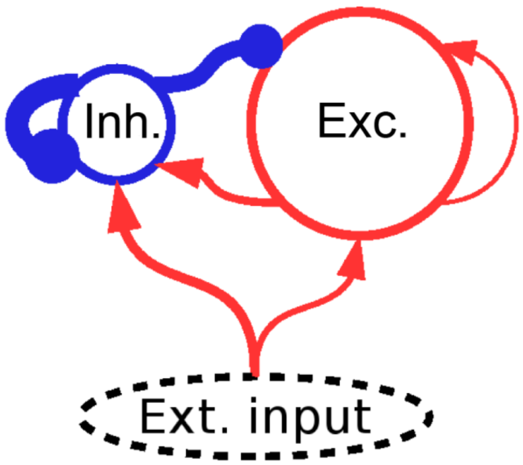

SUPPLEMENTARY FIGURE 1 | Network structure. The network is composed of 1000 inhibitory neurons (blue) and 4000 excitatory neurons (red). Connectivity is random, each directed pair of neurons is connected with a probability of 0.2. The size of the arrows represents schematically the different synaptic strengths. In addition to recurrent interactions both populations receive an external excitatory input. Modified with permission from (Mazzoni et al. 2008).

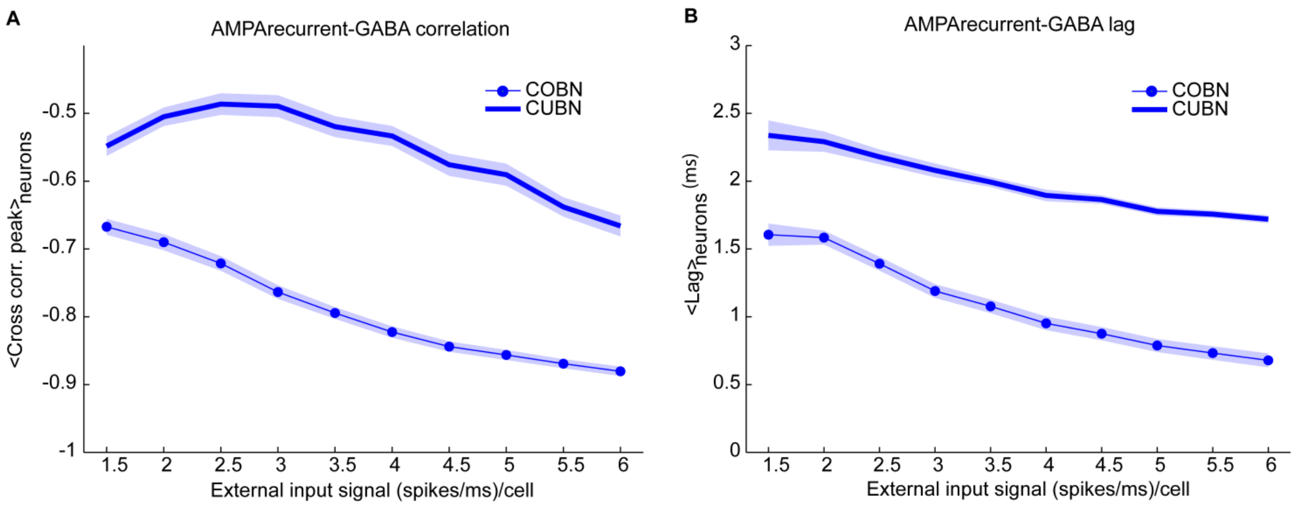

SUPPLEMENTARY FIGURE 2 | Correlation between AMPA and GABA inputs to inhibitory neurons. (A) Same as (Figure 8A) for inhibitory neurons. **(B)** Same as (Figure 8B) for inhibitory neurons**.**

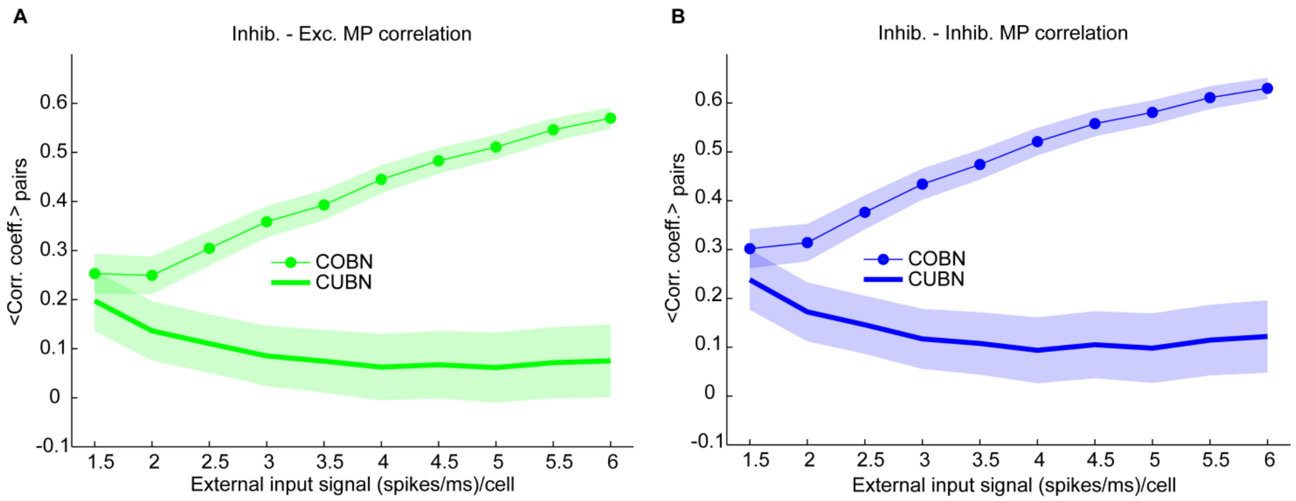

SUPPLEMENTARY FIGURE 3 | Membrane potential correlation across neurons (A) Same as (Figure 9B) for pairs composed by an inhibitory and an excitatory neuron. **(B)** Same as (Figure 9B) for pairs composed by two inhibitory neurons.

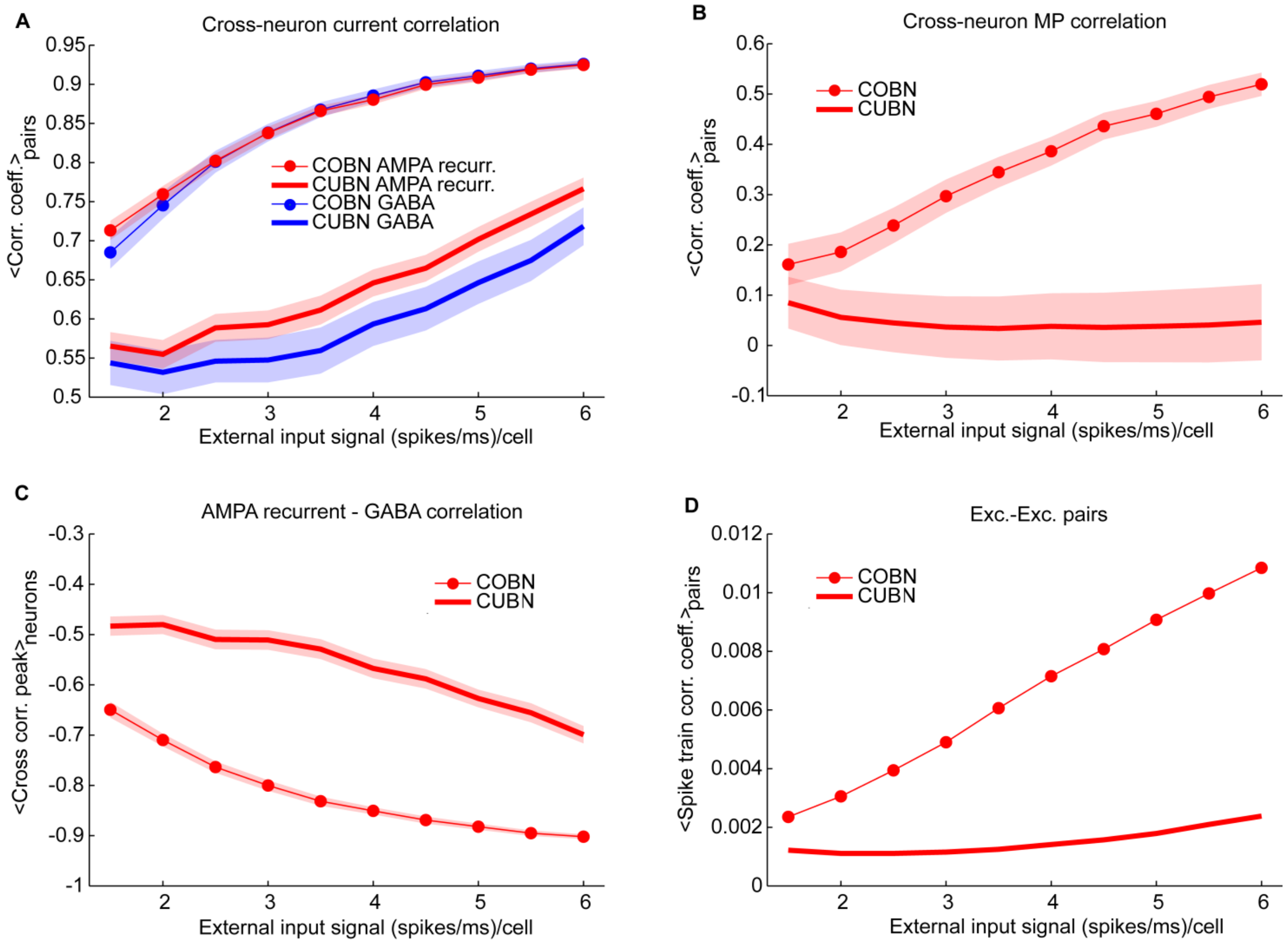

SUPPLEMENTARY FIGURE 4 | Correlations in presence of white noise. Same correlation analysis performed in the main text. The difference lies in the fact that here we model the external input noise, n(t), as a Gaussian white noise instead of an Ornstein Uhlenbeck process (see Methods). The white noise has the same variance of the OU process used in the main text. **(A)** Same as Figure 9A. **(B)** Same as Figure 9B. **(C)** Same as Figure 8A. **(D)** Same as Figure 10A.

Analysis	Variable	Set	Low cond. state (LCS) input: 1.5 spikes/ms		High cond. state (HCS) input: 5 spikes/ms		Focus
			COBN	CUBN	COBN	CUBN	
**First order statistics**	PSP peak amplitude (mV) [Fig. 2F]	GABA on Exc.	0.89±0.02	1.07	0.54±0.03	1.07	PSPs of COBN are smaller than PSPs of CUBN. In COBN, reduction of PSPs from LCS to HCS. (Results from spike triggered averaged considering 300 neurons of the network, see Methods)
		GABA on Inh.	1.09±0.02	1.35	0.65±0.03	1.35	
		Rec. AMPA on Exc.	0.289± 0.001	0.32	0.213± 0.001	0.32	
		Rec. AMPA on Inh.	0.486± 0.003	0.54	0.366± 0.003	0.54	
		Ext. AMPA on Exc.	0.378± 0.002	0.42	0.279± 0.002	0.42	
		Ext. AMPA on Inh.	0.659± 0.003	0.73	0.496± 0.004	0.73	
	τ_{eff} (ms) [Fig. 3A]	Exc.	11.5±0.2	20	3.8±0.2	20	In COBN, reduction of τ_{eff} from LCS to HCS.
		Inh.	6.1±0.1	10	2.2±0.1	10	
	MP (mV) [Fig. 5D]	Exc.	-58.8±0.3	-61.4±0.8	-60.2±0.8	-74±6	COBN MP more stable with input variations than CUBN MP.
		Inh.	-60.0±0.3	-62.0±0.6	-60.7±0.7	-69±4	
	σ_{time}(MP) (mV) [Fig. 5F]	Exc.	2.32±0.07	3.6±0.3	3.06±0.05	8.4±0.8	
		Inh.	2.80±0.06	4.0±0.2	3.87±0.07	8±1	
	FR (Hz) [Fig. 6A]	Exc.	0.45±0.04	0.39±0.03	2.08±0.02	2.08±0.03	Similar FRs between COBN and CUBN
		Inh.	1.2±0.1	1.5±0.1	9.7±0.1	10.6±0.1	
	CV ISI [Fig. 6B]	Exc.	0.98±0.14	0.98±0.16	1.01±0.10	1.14±0.19	CV ISI increases with input in CUBN, while it is constant in COBN.
		Inh.	1.01±0.09	1.02±0.09	1.01±0.04	1.27±0.09	
	Position of gamma peak LFP power (Hz) [Fig. 7A,B]		47±4	44±4	87.3±0.8	87±3	Similar position of LFP gamma peak between COBN and CUBN.
	Mean current $\left\|<\sum_n (I_{syn})>\right\|$ (10^4mV)	Tot. AMPA on Exc.	7.8±0.3	7.6±0.3	28.1±0.2	27.6±0.2	In HCS of COBN, the input currents have fluctuations larger (while the mean values are similar or smaller) than in CUBN.
		GABA on Exc.	3.2±0.3	4.1±0.3	24.0±0.2	28.9±0.3	
	Current fluctuations (10^4 mV) [Fig. 7F]	Tot. AMPA on Exc.	2.8±0.1	2.5±0.1	5.8±0.2	3.4±0.1	
		GABA on Exc.	3.6±0.2	3.8±0.2	12.6±0.3	8.3±0.3	
**Cross correlation peak**	Rec. AMPA-GABA [Fig. 8A,SF2A]	Exc.	-0.73± 0.01	-0.62± 0.02	-0.879± 0.006	-0.62± 0.02	AMPA and GABA currents entering a neuron are more correlated in COBN than in CUBN.
		Inh.	-0.67± 0.01	-0.55± 0.01	-0.856± 0.007	-0.59± 0.02	
**Cross-neuron correlation coefficient**	Rec. AMPA-Rec. AMPA [Fig. 9A]	Exc.-Exc.	0.78±0.01	0.70±0.01	0.914± 0.005	0.70±0.02	Input currents are more correlated across neurons in COBN than in CUBN.
		Inh.-Inh.	0.69±0.01	0.59±0.02	0.898± 0.005	0.66±0.02	
	GABA-GABA [Fig. 9A]	Exc.-Exc.	0.82±0.01	0.76±0.02	0.92±0.01	0.66±0.03	
		Inh.-Inh.	0.82±0.01	0.76±0.02	0.90±0.01	0.66±0.03	
	MP-MP [Fig. 9B,SF3A, SF3B]	Exc.-Exc.	0.24±0.04	0.19±0.06	0.48±0.02	0.05±0.07	MP and Spike Train correlation across neurons increase with input in COBN, while they are constant in CUBN.
		Inh.-Inh.	0.30±0.03	0.24±0.04	0.58±0.02	0.10±0.05	
		Inh.-Exc.	0.25±0.03	0.20±0.05	0.51±0.02	0.06±0.06	
	Sp.Tr.-Sp.Tr. (10^{-2}) [Fig. 10A-C]	Exc.-Exc.	0.4±1.1	0.2±1.0	0.9±1.1	0.2±0.9	
		Inh.-Inh.	1.3±1.3	1.1±1.1	4.7±1.8	1.4±1.4	
		Inh.-Exc.	0.6±1.2	0.4±1.0	1.9±1.3	0.5±1.0	

SUPPLEMENTARY TABLE 1 | Summary of differences between two comparable COBN and CUBN models

This table summarizes our main findings by comparing the values of different features in the COBN used as reference (see Table 2) and in the CUBN, when using two constant stimuli: ν_0=1.5 and 5 (spikes/ms)/cell. These inputs cause respectively a low-conductance state (LCS) and a high-conductance state (HCS). Values are reported as mean ± standard deviations. **PSP peak amplitudes** of the COBN are computed by using a spike triggered averaged over 300 neurons from the network in a simulation of 10.5 s (see Methods). The effective membrane time constant of the COBN, **τ_{eff}**, the membrane potential, **MP**, the fluctuations on time of the membrane potential, **σ_{time}(MP)** and the coefficient of variation of the ISI**, CV ISI**, are computed for each neuron and then averaged across neurons by using data from a single trial (of 10.5 s for τ_{eff}, MP and σ_{time}(MP) and of 100.5 s for CV ISI); the standard deviations are computed across neurons. The average firing rate, **FR**, the **position of the gamma peak of the LFP power** spectrum, the **current mean** and the **current fluctuations** are computed for each trial (of 4.5 s) considering the activity of all the (excitatory or inhibitory) neurons of the network and then are averaged over 50 trials (the standard deviations are computed thus across trials). The current mean and the current fluctuations refer to the sum of the (AMPA or GABA) currents entering all the excitatory neurons, as indicated by the summation over neurons, $\sum_n$, which are exactly the variables used to simulate the LFP. The sum of external AMPA (Ext. AMPA) and recurrent AMPA (Rec. AMPA) is stated as Tot. AMPA. Correlations are computed by using a single trial of 10.5 s. In particular, the **cross correlation peak** is averaged over the neurons obtained from two randomly selected subpopulations of 200 excitatory and inhibitory neurons (see Method), while the **cross-neuron correlation coefficient** is averaged over all the couples of neurons obtained from the same subpopulations (see Methods).
